# Definition of Synovial Mesenchymal Stem Cells for Meniscus Regeneration by the Mechanism of Action and General Amp1200 Gene Expression

**DOI:** 10.3390/ijms251910510

**Published:** 2024-09-29

**Authors:** Kentaro Nakamura, Tsukasa Kitahashi, Ryo Kogawa, Yuichi Yoshino, Izumi Ogura

**Affiliations:** Bioscience & Engineering Laboratory, FUJIFILM Corporation, Ashigarakamigun 258-8577, Kanagawa, Japan; tsukasa.kitahashi@fujifilm.com (T.K.); ryo.kogawa@fujifilm.com (R.K.); yuichi.yoshino@fujifilm.com (Y.Y.); izumi.ogura@fujifilm.com (I.O.)

**Keywords:** mesenchymal stem cells, quality control, critical quality attributes, molecular mechanisms of pharmacological action, synovium

## Abstract

The quality control (QC) of pharmaceutical-grade cell-therapy products, such as mesenchymal stem cells (MSCs), is challenging. Attempts to develop such products have been hampered by difficulties defining cell-type-specific characteristics and therapeutic mechanisms of action (MoAs). Although we have developed a cell therapy product, FF-31501, consisting of human synovial MSCs (SyMSCs), it was difficult to find specific markers for SyMSCs and to define the cells separately from other MSCs. The purpose of this study was to create a method for identifying and defining SyMSCs from other tissue-derived MSCs and to delve deeper into the mechanism of action of SyMSC-induced meniscus regeneration. Specifically, as a cell-type-dependent approach, we constructed a set of 1143 genes (Amp1200) reported to be associated with MSCs and established a method to evaluate them by correlating gene expression patterns. As a result, it was possible to define SyMSCs separately from other tissue-derived MSCs and non-MSCs. In addition, the gene expression analysis also highlighted TNSF-15. The in vivo rat model of meniscus injury found TNSF-15 to be an essential molecule for meniscus regeneration via SyMSC administration. This molecule and previously reported MoA molecules allowed an MoA-dependent approach to define the mechanism of action for SyMSCs. Therefore, SyMSCs for meniscus regeneration were defined by means of two approaches: the method to separate them from other MSCs and the identification of the MoA molecules. These approaches would be useful for the QC of cell therapy products.

## 1. Introduction

Mesenchymal stem cells (MSCs), which are tissue-derived somatic stem cells, have been widely studied in preclinical research and clinical trials, demonstrating various useful differentiation capacities and paracrine effects [1,2,3,4,5,6]. Many studies have been published on the efficacy of MSCs in a wide variety of diseases, including the immunosuppression of graft versus host disease, for which MSCs have been approved [7,8]. Nevertheless, there has been much debate about the usefulness of MSCs, and it has become clear that the quality control (QC) and cell characterization of MSC-based cell therapies are challenging issues [9,10,11]. Although CMC discussions regarding Remestemcel-L have been conducted by the FDA regarding potency and mechanisms of action, there is currently no such product approved in the U.S. by the FDA as of yet [12].

MSCs are known to be composed of crude cell populations rather than monoclonal cells, so they are difficult to define in detail and to control in terms of quality [13]. Furthermore, MSCs have different characteristics in different tissues, but it is difficult to find tissue-specific markers. Overall, clinical outcomes with MSCs tend to be highly variable and there is continued debate over their therapeutic effectiveness, but several MSCs have been successfully approved as cell-therapy products [14,15]. Also, the research on MSC-derived exosome therapy as a derivative clinical application is also underway [16].

Although MSCs themselves are considered effective, the use of QC strategies based on efficacy, safety, stability, and cellular characteristics remains important to ensure the value of all cell-therapy products [17]. This is similar to small-molecule pharmaceutical products [18]. The QC of pharmaceutical products has shifted from quality by testing to quality by design, wherein the establishment of QC items such as quality target product profiles and critical quality attributes (CQAs) is included in the overall design [19].

QC parameters that are relatively easy to evaluate in therapeutic cells include the following: cell count (dose), viability, potency/titer (efficacy), cell surface antigen expression (purity), aseptic processing, mycoplasma negativity, endotoxin level, and tumorigenicity [18]. Minimal criteria published by the International Society for Cellular Therapy can be used to define MSCs specifically [20]. Additionally, tips and tricks for the Good Manufacturing Practice (GMP)-compliant validation of analytical methods for MSCs as medicinal products were reported [21]. QC items that cell-therapy developers consider particularly difficult to navigate include the mechanism of action (MoA), cellular characteristics, equivalence, and homogeneity [22].

Human synovial mesenchymal stem cells (SyMSCs) have been reported to be useful in the treatment of meniscus and knee osteoarthritis and cartilage defects [23,24,25]. We have developed a cell product, FF-31501, composed of human SyMSCs for meniscus tears. The Ph3 clinical trial is currently underway with FUJIFILM Toyama Chemical Co., Ltd. (ClinicalTrials.gov number NCT05777967). In this development, we successfully identified and reported some MoA molecules [26]. On the other hand, we searched for markers specific to synovial tissue but could not find any clear markers as well as MSCs derived from bone marrow and adipose. Therefore, in this study, we created an expression analysis kit called Amp1200, which consists of 1143 genes reported to be associated with MSCs, and attempted to define MSCs derived from synovial tissue by correlating their gene expression patterns. As a result, it was possible to define SyMSCs separately from other tissue-derived MSCs and non-MSCs. A gene, TNSF-15, was also highlighted and was found to be an essential molecule for meniscus regeneration. The scientific significance of this study is that differences that would be buried and overlooked in all gene expression analyses were clearly captured by focusing on a group of genes that are characteristic of MSCs. This led to the differentiation of SyMSCs from other MSCs and the discovery of new mechanistic molecules.

## 2. Results

### 2.1. QC Correlation Method in SyMSCs, Amp1200 Construction of MSC Gene Groups

Unlike a single-molecule drug, a complex, living cell product that has yet to be completely elucidated requires the development of far more specialized QC methods. To this end, we developed a tool called Amp1200, an RT-PCR and amplicon sequencing method that defines the therapy-grade MSC products in MSCs [27]. This was based on the philosophy of “standing on the shoulders of giants”. First, we extracted the gene set that may be involved in MSCs and the functions from the vast accumulated knowledge encompassed in 240 references, 26 commercial gene panels, and 19 classifications of the Gene Ontology (GO) database. This process identified the following: 282 genes involved in immunomodulation, 303 in anti-inflammation, 121 in angiogenesis, 109 in neurogenesis, 119 in cartilage formation, 96 in bone formation, 101 in adipogenesis, 112 in fibrosis suppression, 42 in immunogenicity, 137 in migration, 95 in adhesion, and 113 in senescence; 110 MSC marker genes; and 346 other genes. The total number of genes excluding duplicates was 1199. We excluded genes with overly high expression and for which primers for PCR amplification were not readily available so that we selected 1143 of these genes as being characteristic of MSCs (Table 1 and Table S1).

**Figure 1:**
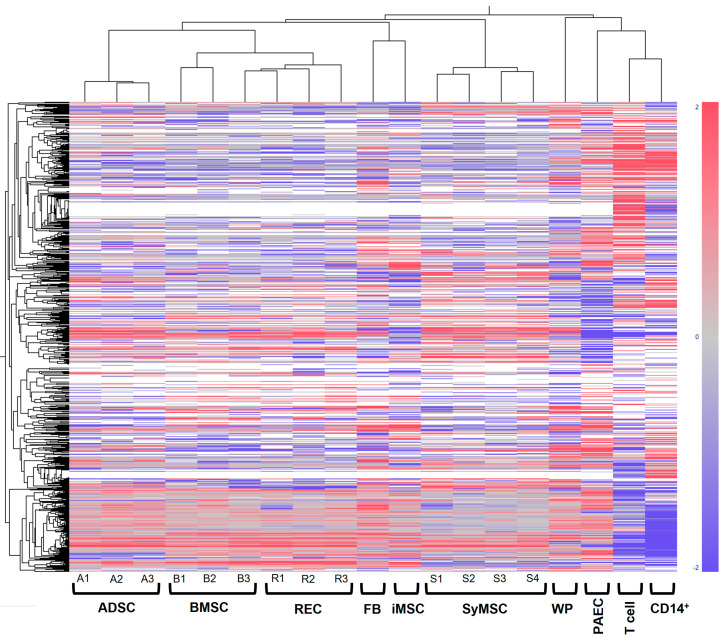
Cell clustering by Amp1200. Lots of SyMSCs (4 donors: S1, S2, S3 and S4), ADSCs (3 donors: A1, A2 and A3), BMSCs (3 donors: B1, B2 and B3), RECs (3 donors: R1, R2 and R3), FBs, iMSCs, WPs, PAECs, T cells, and CD14+ monocytes were clustered by the expression level of genes obtained by Amp1200. The black lines at the top and left showed the results of the clustering. A similar clustering analysis using all 25,193 human genes is shown in Figure S1.

The gene expression data from Amp1200 were used to cluster the following cell types: human SyMSCs, human adipose-derived stem cells (ADSCs), human bone-marrow-derived MSCs (BMSCs), bone-marrow-derived ultra-pure human MSCs (RECs), induced pluripotent stem cell (iPSC)-derived MSCs (iMSCs), human fibroblasts (FBs), human pulmonary artery endothelial cells (PAECs), human white preadipocytes (WPs), human T cells (T cells), and human monocytes (CD14+) (Figure 1). Lot differences among ADSCs, BMSCs, RECs, and SyMSCs were each clustered into a neighborhood and identified as different cell types. The iMSCs were clustered in a neighborhood with FBs and appeared somewhat different from tissue-derived MSCs. CD14+ monocytes, T cells, PAECs, and WPs, which were clearly distinct from MSCs, were clustered separately, whereas FBs were clustered in the vicinity of MSCs.

**Figure 2:**
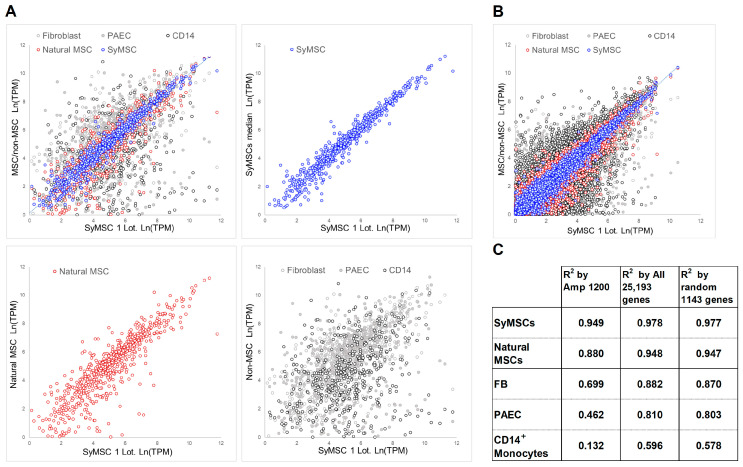
Scatterplot analysis of gene expression in SyMSCs and other cell types. (**A**) Expression of Amp1200-selected genes in one lot of SyMSCs and other cell types, as indicated. (**B**) Expression of all 25,193 human genes in one lot of SyMSCs and MSCs/non-MSCs. (**C**) R^2^ values between one lot of SyMSCs and the indicated cell types based on expression data from the Amp1200-selected (1143 genes), all-human (25,193 genes), and random-human (1143 genes) gene sets.

As the correlation, the coefficient of determination (R^2^) values for each gene group were calculated using the log-treated transcripts per kilobase million (TPM) gene expression values. The numerical R^2^ values for comparisons between each cell population and one SyMSC lot are shown in Table 2 and Figure 2. In the QC of developed MSCs, it is desirable for the R^2^ values between different non-MSC types to be as small as possible and for those between similar MSC types to be moderately large, aiming for significant differences by tissue origin, even within the MSC population. This enables the establishment of boundary values with another MSC population and with the non-MSC population.

The first lot of SyMSCs had an R^2^ of 0.886 with the second lot and an R^2^ of 0.949 with the median for the four lots of SyMSCs. The R^2^ values of BMSCs (0.821), RECs (0.786), and ADSCs (0.821) indicated that the average R^2^ of the other-tissue-derived MSCs is 0.809 ± 0.020 and an R^2^ of ≥0.829 could be used to define SyMSCs as a subpopulation within the MSC population. In addition, the R^2^ values for SyMSCs compared with non-MSC types, such as FBs, WPs, PAECs, T cells, and CD14+, ranged from 0.093 to 0.720, from which a threshold of 0.720 was used to classify and monitor non-MSCs.

Unlike biogenic tissue-derived MSCs, artificially produced iPSC-derived MSCs exhibited a gene expression profile that was similar to FBs, resulting in an R^2^ of 0.663 with SyMSCs. This R^2^ was lower than those of FBs (0.699) and WPs (0.720), suggesting that iPSC-derived MSCs should be classified as a distinct population from natural tissue-derived MSCs.

### 2.2. Expression of Amp1200-Selected Genes, All Human Genes, and 1143 Random Genes in SyMSCs

To verify the significance of Amp1200 gene selection, R^2^ values were recalculated using the expression results for all 25,193 human genes (all-human) and a set of 1143 randomly selected human genes (random-human). As expected, the R^2^ values using the all-human and random-human gene sets were almost equal (Table 2). The coefficient of variation (CV) of the R^2^ for each of the tested cell types with respect to the SyMSC med was calculated as follows: %CV = |(R^2^ SyMSC med − R^2^ other cell type)/R^2^ SyMSC med| × 100. Large CV values indicate that different cell types are distinguishable and point to the high discrimination and detection power of a cell-therapy product. We found that CV values were generally higher for the Amp1200-selected genes than for all-human and random-human gene sets. Specifically, for any given cell type, the CV value was more than twice as high with the Amp1200-selected gene set, indicating that Amp1200 is a suitable tool for MSC QC. Table 2 shows the degree of detection power as ratios of the CVs of the Amp1200, all-human, and random-human gene sets.

### 2.3. SyMSCs Isolated from Human Synovium over Time in Cultures

**Figure 3:**
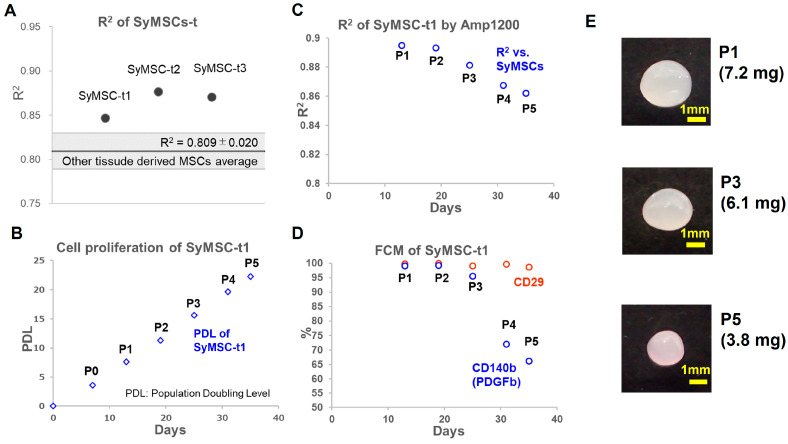
Data of SyMSC-t. (**A**) R^2^ values in SyMSC-t. The criteria, 0.809 ± 0.020, for distinguishing SyMSCs from other tissue-derived MSCs are highlighted. (**B**) Cell proliferation of SyMSC-t1 cultured to P5 (35 days). (**C**) R^2^ values in SyMSC-t1 at P1–P5. (**D**) FCM analysis of CD29 and CD140b (PDGFb) expression in SyMSC-t1 at P1–P5. (**E**) Representative images of cartilage matrix production in the chondrogenic differentiation of SyMSC-t1 at P1, P3, and P5. No image is available because it does not produce a cartilage matrix in the absence of differentiation induction (its weight is less than 0.1 mg).

SyMSCs, referred to as SyMSC-t in this paper, isolated from three human synovial samples collected at Tokyo Medical and Dental University were correlated using Amp1200. As a result, the R^2^ values of the three established cells with SyMSCs were 0.847, 0.876, and 0.871, respectively, all of which exceeded the assumed criterion value of 0.829. In addition, the cell sample with the lowest correlation (SyMSC-t1) was analyzed for changes in behavior over a long period of culturing. Briefly, SyMSC-t1 were seeded directly after digestion of synovial tissue and cultured for 35 days to passage (P)5 and evaluated for proliferation, expression of CD29 and CD140b (also known as platelet-derived growth factor receptor b (PDGFRb)), Amp1200 gene selection, and cartilage matrix production (Figure 3). Flow cytometry (FCM) analysis showed CD29 expression in 95% of cells until P5, but a decreasing trend in CD140b expression over time was observed after P3 (Figure 3D). The evaluation of cartilage matrix production is shown in Figure 3E.

### 2.4. Contribution of the SyMSC-Characteristic Molecule TNFSF-15 to In Vivo Efficacy of Rat SyMSCs

**Figure 4:**
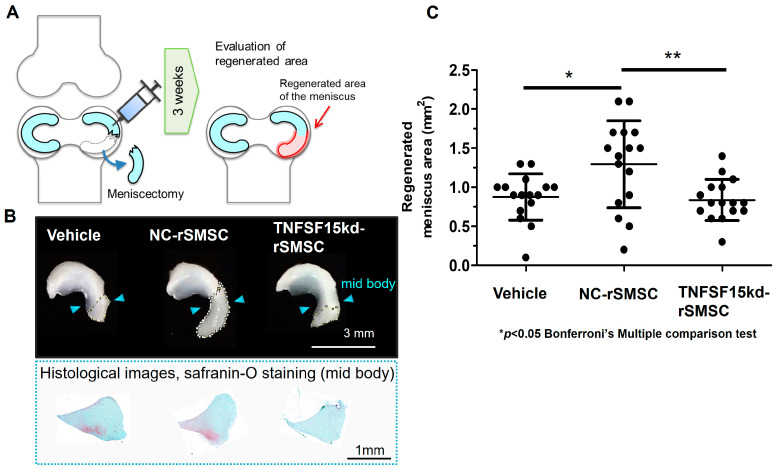
Evaluation of TNFSF-15 in meniscus regeneration in an in vivo rat meniscectomy model. (**A**) Schematic diagram of rat meniscectomy with the administration of siRNA-treated rSMSCs or vehicle and evaluation of the regenerated area 3 weeks later. (**B**) Representative meniscus repair images, macroscopic and safranin-O staining histological images, 3 weeks after injection with the vehicle control, NC-rSMSCs, or TNFSF-15kd-rSMSCs. (**C**) Evaluation of regenerated meniscus area 3 weeks after injection with vehicle control, NC-rSMSCs, or TNFSF-15kd-rSMSCs; * *p* < 0.05, ** *p* < 0.01 analyzed by Bonferroni’s multiple comparison test.

The Amp1200 tool identified TNFSF-15 as a characteristic molecule of SyMSCs that tends to be more highly expressed in SyMSCs than in other cell types. Therefore, we generated rat SyMSCs (rSMSCs) with TNFSF-15 knockdown to test its contribution to post-meniscectomy regeneration using a rat meniscectomy model. At 3 weeks post-meniscectomy (Figure 4A), the injection of a vehicle control resulted in a slight increase in the size of the meniscus attributable to natural healing ability (Figure 4B and Figure S2). While injection of control siRNA-treated ACI rat-derived SyMSCs (rSMSCs), NC-rSMSCs, significantly increased the size of the meniscus compared with the vehicle, injection of TNFSF-15 siRNA-treated rSMSCs, TNFSF-15kd-rSMSCs, led to a significantly reduced effect comparable to that of the vehicle (Figure 4C).

### 2.5. QC Concept for SyMSCs

**Figure 5:**
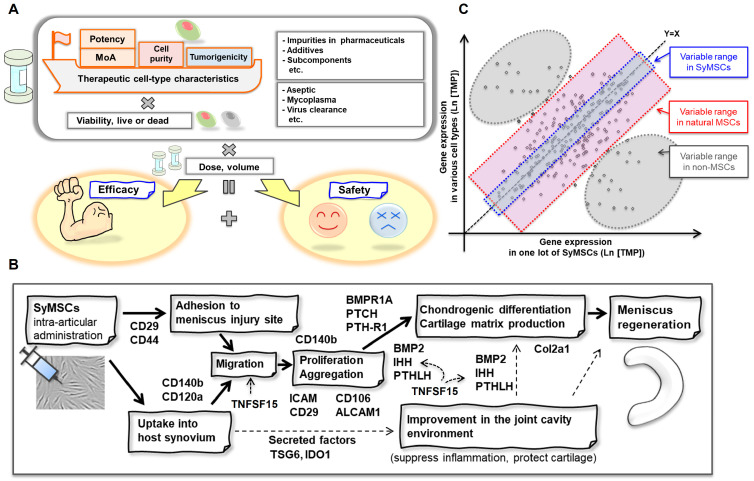
Conceptual design of a two-pronged QC method for cell-therapy products using SyMSCs as a model. (**A**) Schematic diagram of QC concepts important in the development cell-therapy products. (**B**) Summary of molecules involved in the hypothesized MoA of SyMSCs in meniscus regeneration. Solid arrows indicate mechanisms with a high degree of certainty based on cited references, while dotted arrows indicate mechanisms with a low degree of certainty that include speculation. (**C**) Scatterplot of gene expression in SyMSCs and a variety of other cell types to illustrate simultaneous monitoring of many molecules correlated with a cell-therapy product.

Our QC concept for cell-therapy products is illustrated in Figure 5A. We proposed a two-pronged QC method designed to identify key MoA molecules that directly contribute to efficacy (MoA method) and to monitor the cell-therapy product for unexpected changes in cell type (correlation method). The correlation method is a technique that distinguishes between MSCs derived from synovial tissue and MSCs derived from other tissues. A summary of the current understanding of the MoA of SyMSCs in meniscus regeneration is illustrated in Figure 5B [26,28,29,30,31,32,33,34], with a scatterplot comparing gene expression patterns in SyMSCs and a variety of other cell types shown in Figure 5C.

## 3. Discussion

Using SyMSCs as a model, we here describe the design of a two-pronged QC method for the development of cell-therapy products: (1) the cell-type-dependent correlation with many molecules related to the target cell type and (2) the MoA-dependent identification of CQAs.

### 3.1. Cell-Type-Dependent Correlation QC Method

Our hypothesis for cell-type-dependent gene expression patterns as a QC measure was verified using SyMSCs as a model. Using the Amp1200 tool, we extracted a wide range of genes known to be related to MSCs and their characteristics. For ease of comprehension, the expression pattern of this group of genes was evaluated numerically using the R^2^ as a bird’s eye-level indicator, in contrast to methods that rely on the expression of individual genes. Our results demonstrate that it is now possible to clearly distinguish SyMSCs from non-MSCs or MSCs of different tissue origin.

Our findings also reveal that the identification of SyMSCs based on the Amp1200 including 1143 genes with previously established cell-type-specific relatedness was quite sensitive. Indeed, comparison of the Amp1200 gene set with the 1143 random gene set suggested that the use of established cell-type-specific information is of high value in QC of cell-therapy products. It is widely understood that the percentage of variation in human gene expression from cell type to cell type is approximately 5% and, thus, the selection of 1143 MSC-characteristic genes from 25,163 possible genes (~4.5%) using Amp1200 would be appropriate.

Budeus et al. mentioned that gene expression analysis was carried out with the aim of discriminating MSCs from FBs and that 1352 genes were identified [35]. However, it was difficult to explain what the gene set meant for MSCs, and it was only to be used as a gene set to distinguish them from FBs. In the DEG-based gene set selection method based on a single experiment, genes are selected based on differential expression results as a phenomenon based on data restricted by the specific conditions (medium, measurement conditions, environment, etc.) and purpose of the experiment. Therefore, it has the limitation that it cannot fully explain the meaning and relationship of each gene set with MSCs.

On the other hand, as described by Jovic et al., a trend in research over the last few years has been an attempt to identify subpopulations of cells using scRNA-seq [36]. For example, Kanazawa et al. classified bone marrow MSCs into seven subpopulations via scRNA-seq and identified the function of individual subpopulations [37]. Yi et al. used scRNS-seq to classify MSCs derived from different tissue sources—AMMSCs, UCMSCs, and CMMSCs—into six subgroups and to identify what functional characteristics each tissue-derived MSC had as a subpopulation [38]. They stated that this could enable the selection of MSCs suitable for therapeutic targets. Subpopulation identification by means of scRNS-seq is useful for the purpose of identifying specific genes expressed by subpopulations. However, its use for product quality control, including its usage and positioning, has not yet been established. Therefore, this study used conventional RNA-seq, a widely used technique. scRNA-seq-based product quality control will be established in the future.

Similar to our study, attempts to classify stem cells using conventional RNA-seq have been made in studies such as Yamatani et al. [39]. In this report, gene clusters for classifying iPSCs, HSCs, and MSCs were extracted by means of exhaustive microarray and DEG analysis and, then, changes in each stem cell were evaluated for each tissue of origin. As a result, they stated that they had identified a group of genes characteristic of MSCs. However, it was stated that the expression of these gene groups showed almost no differences between MSCs’ tissue of origin. The usual process of finding gene groups from the results of a single experiment based on DEGs involves selecting gene groups based on data influenced by certain experimental conditions (medium, measurement conditions, environment, etc.). It is therefore characterized by an inability to explain the importance and value of each gene in MSCs. In contrast, we used a method whereby the importance and value of each gene for MSC were sought from a wide variety of predecessor report groups, based on which the gene groups were predefined as Amp1200, and the tested and expression differences were assessed by pattern correlation. This allowed the relationship with MSCs for each gene to be explained on the basis of previous studies. In addition, the resulting convergence of the results accumulated by a number of researchers resulted in our more acute observation of tissue-derived differences in MSCs.

Culture results for newly isolated SyMSC-t from a human patient suggested that the R^2^ of Amp1200-selected genes could be useful in the QC process during the manufacturing of cell-therapy products. Until P5, the population of SyMSC-t did not deviate from the cell type, with the slight decrease in R^2^ over time capturing signs of change. Additionally, it was indicated that the protein levels of MoA players CD140b and PDGFb were decreased from P3 onwards, while cartilage matrix production, considered to represent potency, was reduced at P5. These indications support the hypothesized concept of a collection of MoA molecules comprising the foundation of cell-specific characteristics, with their effects on potency observable as a phenotype. Although autologous cell-therapy products are thought to be unaffected by potency and cell-type purity because they do not involve extended passaging, the detection of slight changes in the potency and cell-type purity becomes important for allogeneic cell-therapy products that are cultured in large quantities.

### 3.2. MoA-Dependent QC Method

Kitahashi et al. [26] evaluated six candidate molecules involved in the MoA of SyMSC action in meniscus regeneration: CD29 (integrin β1), CD140b (PDGFRβ), CD44, CD106 (Vcam1), CD120a (Tnfr1), and Col2a1. Three of these, CD29, CD140b, and Col2a1, were found to be necessary for efficacy in a rat meniscectomy model in this report. Because the contributions of CD29 and CD140b to in vivo efficacy were found to be remarkably strong, we also monitored them in the development of FF-31501 (ClinicalTrials.gov number NCT05777967). Our knockdown experiments in a rat meniscectomy model in this study further identified the importance of TNFSF-15 in meniscus regeneration. TNFSF-15, also known as TL1A, is a well-known inflammatory mediator in rheumatoid arthritis [40,41,42]. Patients with rheumatoid arthritis reportedly exhibit elevated TL1A levels in the joint cavity [43]. However, TL1A has also been shown to induce migration of synovial cells and increase the expression of Indian Hedgehog (IHH) and its receptor Patched (PTCH) [44]. Our finding that TNFSF-15 was essential for meniscal repair implied that the role of TNFSF-15 may not be limited to that of a simple inflammatory marker. Rather, trace expression of TNFS15 may function as a key mediator in IHH and PTCH signaling, which induces synovial cell migration and leads to chondrogenic differentiation, cartilage matrix production, and protection pathways.

It should be noted that the results of this study were based on a limited number of genes, and the robustness of this QC method and its suitability for validation should be examined in future studies. Furthermore, apart from MoA-dependent and cell-type-dependent QC, a wide variety of other efficacy-related properties of cell-therapy products must be controlled, including safety, potency, titer, cell count, and viability. Single-cell analysis technologies have undergone dramatic developments and can now be used inexpensively to monitor such parameters. In the future, rather than viewing cell-therapy products as crude populations, they will be managed as subpopulations that form new, desired populations of therapeutic cells.

Although this study was focused on the development of a QC method for cell-therapy products, there are many combination products of cells and biomaterials under development [45]. Our team is also conducting research on autologous insulin-producing cell-based agents for type 1 diabetes and autologous MSC-based agents for stroke, and investigator-initiated clinical trials of combination products of MSCs and biomaterials are underway [46,47,48,49]. Such combination products will require the consideration of cell-specific characterization and QC as applied research problems. We anticipate that the cell-therapy modality will become increasingly more common in the treatment of a wide range of conditions and diseases.

## 4. Materials and Methods

### 4.1. Identification of MoA Molecules

Molecules involved in the MoA of SyMSC product efficacy in meniscus regeneration were targeted in a similar manner to any other pharmaceutical product. Specifically, as previously described [26], we selected key hypothesized MoA candidate molecules expressed in MSCs: CD29, CD44, CD106, CD120a, CD140b, and Col2. SyMSCs were derived from rats with knockout or knockdown of each of these candidate molecules and evaluated for efficacy in a rat meniscectomy model. Those with significantly reduced efficacy were identified as MoA molecules.

### 4.2. Preparation of Amp1200 for MSCs

On the basis of previous studies of MSCs, encompassing 240 references, 26 commercial gene panels, and 19 classifications from the GO database, we extracted a set of genes that may be related to MSC functions and characteristics. These references, panel information, and database information are provided in Supplementary Materials Document S1. The Amp1200 gene set was created by removing duplicate genes, those expressed at too high a level for amplicon sequencing, and those for which primers were not readily available.

### 4.3. MSC Culture and RNA Preparation

Human SyMSCs (Articular Engineering, Northbrook, IL, USA, CDD-H-2910-N), human ADSCs (Lonza, Basel, Switzerland, PT-5006), human BMSCs (Lonza, Basel, Switzerland, PT-2501), human RECs (PuREC, Izumo, Japan, 387-16591), and iMSCs (FUJIFILM Cellular Dynamics, Madison, WI, USA, iCell MSC R1098) were all cultured in MEMα (FUJIFILM Wako Pure Chemical Corporation, Osaka, Japan. 289-3365) containing 20 μg/mL gentamicin sulfate (Takada, Saitama, Japan, Gentamicin Injection 60) and 15% *v*/*v* fetal bovine serum (FBS; Selborne, Alton, Hampshire, UK, FBS-04). Frozen cells were thawed at 37 °C, centrifuged at 200× *g* for 5 min, resuspended in a medium for seeding into flasks at 0.25–0.50 × 10^4^ cells/cm^2^, and cultured at 37 °C in a 5% CO_2_ atmosphere. RNA was extracted from the cells after two passages. A 0.05% solution of trypsin and ethylenediaminetetraacetic acid (Thermo Fisher Scientific, Waltham, MA, USA, 25300) was used for cell detachment. RNA extraction was performed using QIAGEN RNeasy kits (QIAGEN, Tokyo, Japan, 74104).

### 4.4. Non-MSC Culture and RNA Preparation

Human FBs (Lonza, Basel, Switzerland, CC-2509), human PAECs (ATCC, Manassas, VA, USA, PCS-100022), human WPs (PromoCell, Heidelberg, Germany, C-12732), human T cells (Lonza, Basel, Switzerland, 2W-200), and human CD14^+^ monocytes (PromoCell, Heidelberg, Germany, C-12909) were cultured as follows. FBs, PAECs, WPs, and CD14^+^ monocytes were thawed in frozen vials at 37 °C, to which 10 mL of MEMα containing 15% FBS and 20 µg/mL gentamycin was added slowly, followed by centrifugation at 300× *g* for 5 min at room temperature and removal of the supernatant. T cells were thawed at 37 °C, to which the following solution was slowly added: 1 mL of RPMI medium containing NEAA, 1 mmol/L sodium pyruvate, 2 mmol/L L-alanine and L-glutamine, 10 mmol/L HEPES, 10% FBS, and 20 U/mL DNase I. After the mixture was centrifuged at 300× *g* for 5 min at room temperature, the lysate was prepared by removing the supernatant and replacing lysis buffer containing QIAGEN RNeasy (QIAGEN, Tokyo, Japan, 74104), RLT Buffer, and DTT (Wako, Osaka, Japan, 047-08973). The lysate was used for RNA extraction. RNA extraction was performed using QIAGEN RNeasy kits.

### 4.5. Gene Library Construction, Quantitative PCR (qPCR), and Sequencing

Reverse transcription was performed using Ampliseq cDNA Synthesis for Illumina kits (Illumina, San Diego, CA, USA, 20022654), in accordance with the manufacturer’s protocol. The first round of PCR was then performed using AmpliSeq for Illumina Custom RNA Panel (Illumina, San Diego, CA, USA, 20020496) and AmpliSeq for Illumina Library PLUS (Illumina, San Diego, CA, USA, 20019102). Next, partial digestion of the amplicons was performed using AmpliSeq for Illumina Library PLUS (Illumina, San Diego, CA, USA, 20019102). For the ligation of index adapters, AmpliSeq for Illumina Library PLUS (Illumina, San Diego, CA, USA, 20019102) and AmpliSeq for Illumina CD Indexes Set A (Illumina, San Diego, CA, USA, 20029105) were used. The supernatant was collected and used as the next-generation sequencing (NGS) library, which was adjusted for concentration and mixed with KAPA Master Mix (KAPA BIOSYSTEMS, Wilmington, MA, USA). qPCR was performed on a CFX96 Real-Time System and products were quantified. For sequencing, the PhiX Control kit v3 (Illumina, San Diego, CA, USA, TG-110-3001) was used. The NGS library was diluted, denatured to single-stranded DNA via the addition of 0.2 N NaOH solution in HT1 buffer, and added to the reagent cartridges of the kit and sequenced on a MiSeq (Illumina, San Diego, CA, USA).

### 4.6. Data Processing

Read data imported into QIAGEN CLC Genomics Workbench software ver.20 were converted into Phred quality scores, which were obtained by taking the log of the error probability of a nucleotide and multiplying it by −10. Using the Phred score, quality trimming was performed as follows: (1) the Phred score was converted to a *p* value; (2) the difference between the limit (0.05) set at the time of trimming and the *p* value was calculated; and (3) the cumulative sum of the differences was calculated, where values ≤ 0 were set to 0. The read start point after trimming was the point where the cumulative sum first became >0, and the read end point after trimming was the point where the cumulative sum was greatest. After quality trimming, read counts were converted to expression levels by aligning read sequences with the most similar regions in the genome, from which reads per kilobase million (RPKM) units were calculated. The TPM was calculated by dividing the RPKM by the sum of the RPKMs. When the TPM values were processed logarithmically, missing values were inserted with values below the minimum measured value for QC purposes to give weight to the lack of expression. Clustering and processing were performed using the Subio platform (ver. 1.24.5859, Subio Inc., Aichi, Japan).

### 4.7. Expression Analysis of All Human Genes

Human transcriptome sequencing analysis was performed on extracted cellular RNA at RIKEN GENESIS, Inc. (Kawasaki, Japan). After sequencing the samples on a NovaSeq6000 (Illumina, San Diego, CA, USA. 20012850), read data were analyzed using DRAGEN ver. 3.7.5 to calculate the gene expression levels of all 25,193 human genes as TPMs.

### 4.8. Human Synovial MSC Isolation and Culture, and Ethics Approval

The procedures involving human cells were performed in accordance with the standards of the Declaration of Helsinki (1989). Informed consent was obtained from the study participant. The protocol was approved by the Medical Research Ethics Committee of Tokyo Medical and Dental University (approval no. M2017-142) and the Human Research Ethics Committee of Fujifilm corporation (approval no. #190-A). The confidentiality of the donor was maintained. All methods were performed in accordance with the relevant guidelines and regulations.

Human synovium was harvested from the knee of an OA patient during total knee arthroplasty. SyMSC-t were isolated from the synovium in accordance with the methods previously described [50]. Briefly, cell suspensions enzymatically treated with collagenase were seeded into flasks at 0.10 × 10^4^ cells/cm^2^ and cultured in MEMα containing 20 μg/mL gentamicin sulfate and 20% *v*/*v* FBS for 35 days at 37 °C in a 5% CO_2_ atmosphere. The cells were passaged every 6–7 days. Cell proliferation, FCM analysis of CD29^+^ and CD140b^+^ cells, the expression of Amp1200-selected genes, and chondrogenic differentiation were evaluated.

### 4.9. Rat Experiments and Ethics Approval

All animal care and experiments were performed in accordance with the Animal Research: Reporting of in vivo Experiments (ARRIVE) guidelines and the institutional guidelines of the Institutional Animal Care and Use Committee (IACUC) of Shonan Health Innovation Park and were approved by the IACUC (reference number: AU-00040284). To establish rat synovial cells, 10 male ACI rats (age: 3 weeks) were purchased from Japan SLC Inc. (Hamamatsu, Japan). For the meniscus regeneration experiment, 25 female Lewis rats (age: 11 weeks) were purchased from The Jackson Laboratory Japan, Inc. (Yokohama, Japan). All rats were allowed free access to food, water, and activity and were maintained under a 12 h dark–light cycle at a controlled temperature (20–26 °C) and humidity (40–70%).

### 4.10. TNFSF-15 Knockdown in rSMSCs

ACI rat-derived rSMSCs were established by means of the method previously described [26]. Rats were euthanized by bleeding from the inferior vena cava under isoflurane anesthesia. The synovium was harvested from the infrapatellar fat pad in both knees of 10 rats, pooled together, and minced. The minced synovium was then digested with 0.3% Collagenase V solution (Merck KGaA, Darmstadt, Germany) in a water bath at 37 °C for 2 h. The cells were cultured in α-minimum essential medium (αMEM; Thermo Fisher Scientific, Waltham, MA, USA) with 20% fetal bovine serum (FBS; Thermo Fisher Scientific, Waltham, MA, USA) for 8 days under conditions of 5% CO_2_ and 37 °C. The cells were then harvested and were seeded into T225 flasks (Thermo Fisher Scientific, Waltham, MA, USA, 159934) at a density of 1000 cells/cm^2^. On day 4 of the culture, the cells were treated with Silencer™ Select rat TNFSF-15 small interfering (si)RNA ID s141123 (Thermo Fisher Scientific, Waltham, MA, USA, 4390816) or negative control siRNA (Thermo Fisher Scientific, Waltham, MA, USA, 4390844) for 3 days, generating TNFSF-15kd-rSMSCs and NC-rSMSCs, respectively. On day 7, the cells were collected, and frozen stock was prepared at 2 × 10^8^ cells/mL in cell preservation solution CP-1 High Grade (Kyokuto Pharmaceutical Industrial Co., Ltd., Tokyo, Japan, 27207). The cells were then stored at −150 °C until injection into experimental animals.

### 4.11. Meniscectomy and Transplantation of TNSF15-Knockdown Cells

As previously described [26], female rats underwent bilateral knee meniscectomy under isoflurane anesthesia. Female rats were anesthetized with isoflurane. Both the right and left knee joints underwent surgery. A medial parapatellar incision and lateral dislocation of the patellar tendon were conducted to expose the medial meniscus. The anterior insertional ligament of the medial meniscus was transected to dislocate the medial meniscus anteriorly, and the medial meniscus was resected at the level of the medial collateral ligament. The capsule was closed using nylon sutures, and the vehicle (CP-1 High Grade), TNFSF-15kd-rSMSCs, or NC-rSMSCs (1 × 10^7^ cells/50 μL) were injected into the knee joint using a 28G needle. The knee joint was moved three times and the skin was sutured. The time required was approximately 40 min per animal for both knees. Eight rats per condition were used for the knockdown experiments and were allowed to walk freely in their cages after the surgery. Three weeks after the MSC injection, rats were euthanized via exsanguination by cutting the thoracic aorta under isoflurane anesthesia. Then, the knee joint was removed and the medial meniscus was photographed. The regenerated area of the meniscus was measured with ImageJ (1.54 g, National Institutes of Health, Bethesda, MD, USA).

### 4.12. Statistics

All the group comparisons of in vivo experiments were performed using one-way analysis of variance with Bonferroni’s multiple comparison test. Statistical tests were conducted using GraphPad Prism (v5.04, GraphPad Software Inc., San Diego, CA, USA). A *p* value < 0.05 was considered to indicate statistical significance.

## Figures and Tables

**Table 1:** MSC characteristic genes (1143) selected by Amp1200.

GO Functions	Number of Genes	Representative Genes Related to MSC Function ^a^
Immunoregulation	282	*IDO1*, *IDO2*, *PTGES*, *TGFB1*, *HGF*
Anti-inflammatory	303	*PTGS2*, *IDO1*, *NOS2*, *TGFB1*, *TNFAIP6*
Angiogenesis	121	*VEGFA*, *TGFB1*, *CCL2*, *ANGPT1*, *FGF2*
Neurogenesis	109	*BDNF*, *GDNF*, *VEGFA*, *NGF*, *NTF3*
Osteogenesis	96	*CBFA1*, *ALPL*, *COL1A1*, *BGLAP*, *SPP1*
Adipogenesis	101	*PPARG*, *FABP4*, *CEBPA*, *LPL*, *ADIPOQ*
Chondrogenesis	119	*COL2A1*, *ACAN*, *SOX9*, *COL10A1*, *COL1A1*
Fibrosis	112	*HGF*, *VEGFA*, *IL10*, *ACTA2*, *PGE2*
Immunogenicity	42	*CD274*, *CD40*, *CD80*, *CD86*, *TNFSF14*
Migration	137	*CXCR4*, *CCR1*, *CCR2*, *MMP2*, *CXCR6*
Adhesion	95	*ITGA4*, *ITGB1*, *VCAM1*, *ICAM1*, *ITGA5*
Senescence	113	*CDKN2A*, *GLB1*, *CDKN1A*, *TRP53*, *HAS1*
MSC selection markers	110	*EGF*, *CD19*, *ZFP42*, *NOTCH1*, *TGFB3*
Others	346	*CCL2*, *IL6*, *VEGFA*, *CXCL12*, *IL8*
Total	1199	
Excluded genes	18	
	38	Genes without readily available PCR primers
Amp 1200	1143	High-expression genes

^a^ Additional details and the symbols and NCBI numbers of all 1143 genes are provided in Table S1.

**Table 2:** Comparison of R^2^ and detection power of the Amp1200-selected, all-human, and random-human gene sets in SyMSCs and different cell types.

			Amp1200 Gene Set R^2^	SyMSC CV	Random-Human Gene Set R^2^	SyMSC CV	All-Human Gene Set R^2^	SyMSC CV	Detection Power (Amp1200 vs. Random- Human)	Detection Power (Amp1200 vs. All-Human)
**MSCs**	Natural MSCs	Secondary lot of SyMSCs	0.886	7%	0.951	3%	0.947	3%	2.47	2.07
		SyMSCs med	0.949	0%	0.977	0%	0.978	0%	N/A	N/A
		BMSCs, med	0.821	14%	0.931	5%	0.928	5%	2.88	2.62
		RECs, med	0.786	17%	0.913	7%	0.912	7%	2.63	2.56
		ADSCs, med	0.821	13%	0.917	6%	0.919	6%	2.19	2.24
	Artificial MSCs	iMSCs	0.663	30%	0.859	12%	0.866	11%	2.49	2.63
**Non-MSCs**		FBs	0.699	26%	0.870	11%	0.882	10%	2.40	2.67
		WPs	0.720	24%	0.890	9%	0.886	9%	2.71	2.58
		PAECs	0.462	51%	0.803	18%	0.810	17%	2.88	2.99
		CD14^+^ monocytes	0.132	86%	0.578	41%	0.596	39%	2.11	2.20
		T cells	0.100	89%	0.541	45%	0.592	39%	2.00	2.27

ADSCs = human adipose-derived stem cells; BMSCs = bone-marrow-derived MSCs; CV = coefficient of variation; FBs = human fibroblasts; iMSCs = induced pluripotent stem cell (iPSC)-derived MSCs; med = median value; MSC = mesenchymal stem cell; PAECs = human pulmonary artery endothelial cells; RECs = bone-marrow-derived ultra-pure human MSCs; SyMSCs = synovial MSCs; WPs = human white preadipocytes.

## Data Availability

The Amp 1200 gene list is described in Supplemental Table S1 and the gene expression data are stored in Supplemental Table S2. Any other additional data and information are available from the corresponding author upon reasonable request.

## Supplementary Materials

The following supporting information can be downloaded at: https://www.mdpi.com/article/10.3390/ijms251910510/s1.
