# Supplementary material for: Definition of Synovial Mesenchymal Stem Cells for Meniscus Regeneration by the Mechanism of Action and General Amp1200 Gene Expression
Source: Int J Mol Sci. 2024 Sep 29;25(19):10510. doi: 10.3390/ijms251910510 (PMC11476826; doi:10.3390/ijms251910510)
Supplement: Supplementary file 1 [file ijms-25-10510-s001.zip › Supplemental _FigS1_S2_DocS1.pdf]

Supplemental Figure S1. Clustering by the expression of 25,193 genes

SyMSCs, ADSCs, BMSCs, RECs, FB, iMSC, WP, PAEC, T cell and CD14^{+} were clustered by the expression of 25,193 genes. iMSC was clustered closer to FB than the tissue-derived native MSCs.

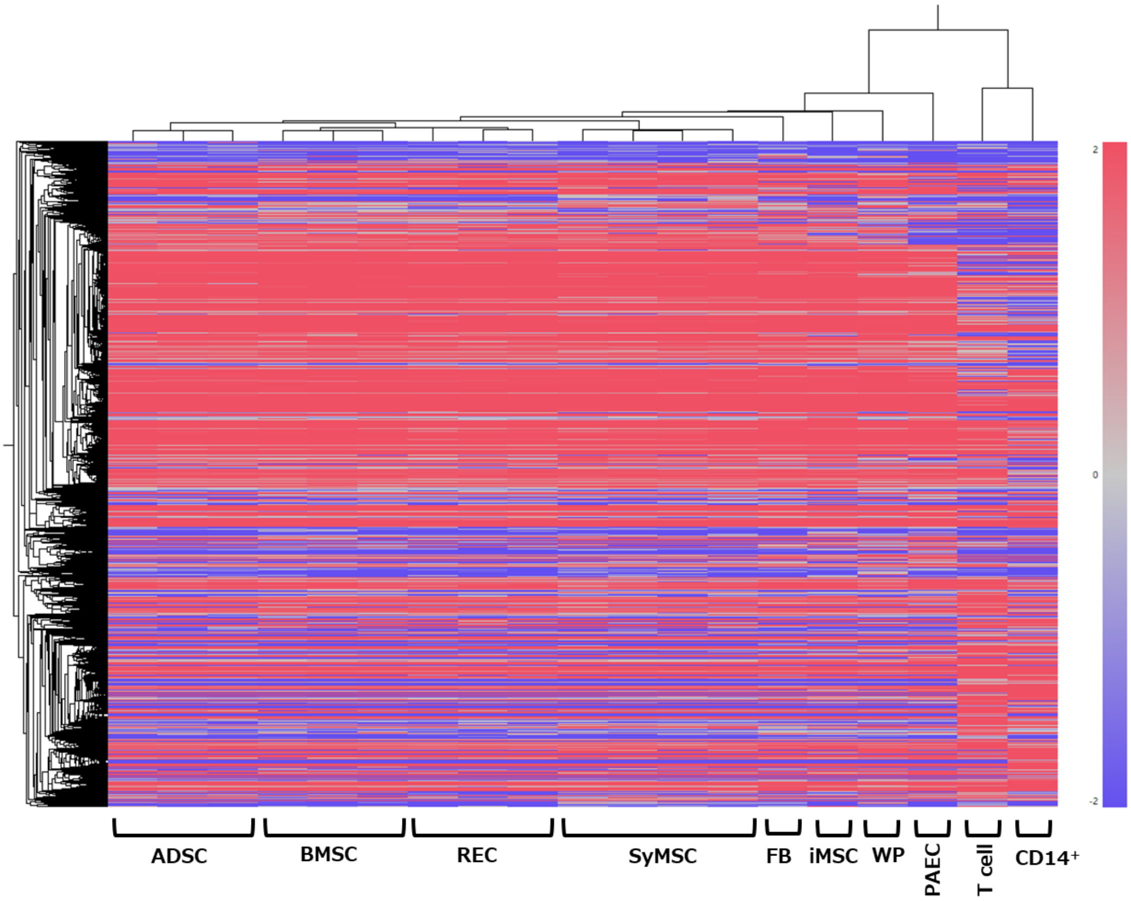

Supplemental Figure S2. Images of the regenerated meniscus in a rat meniscectomy model.

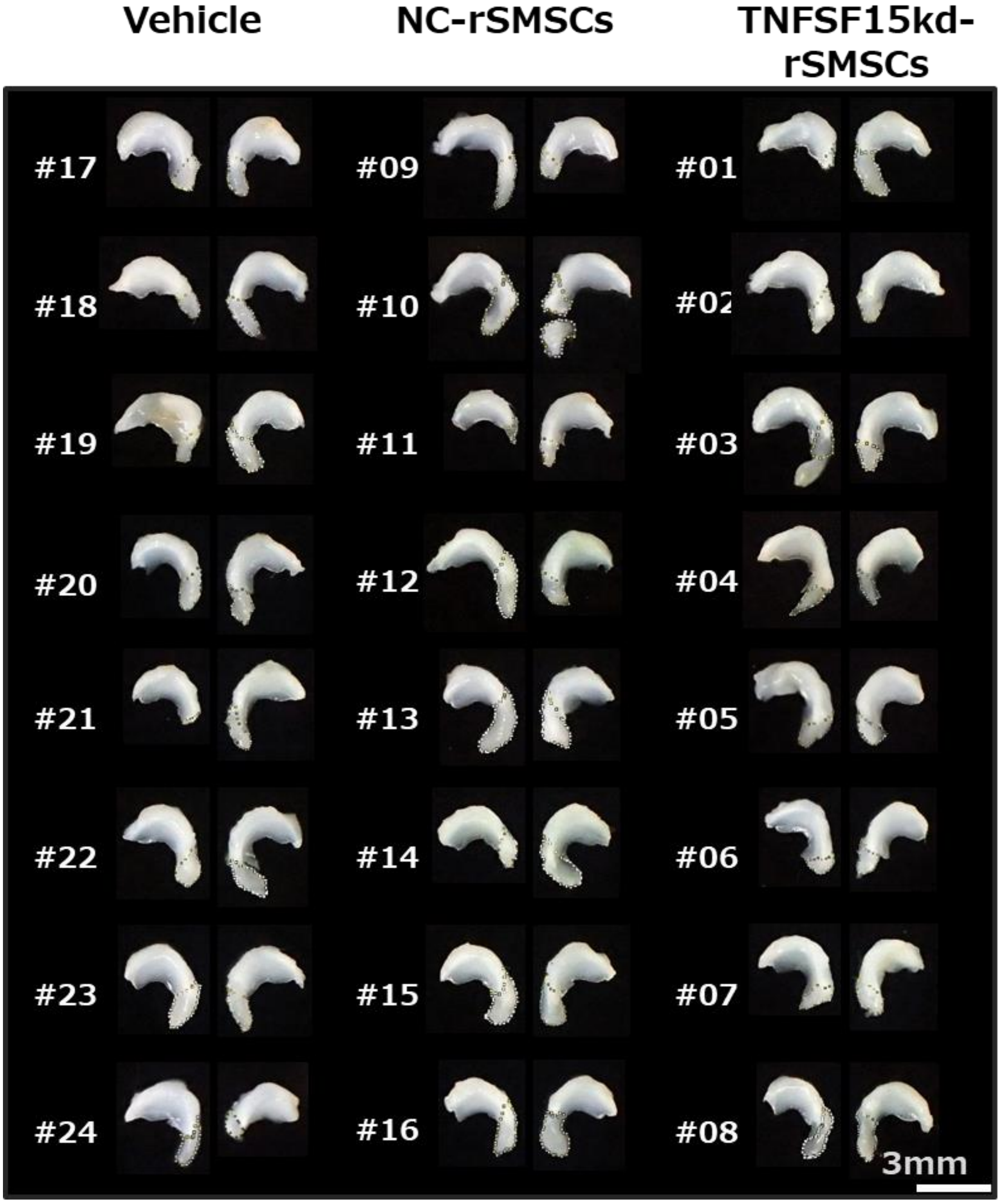

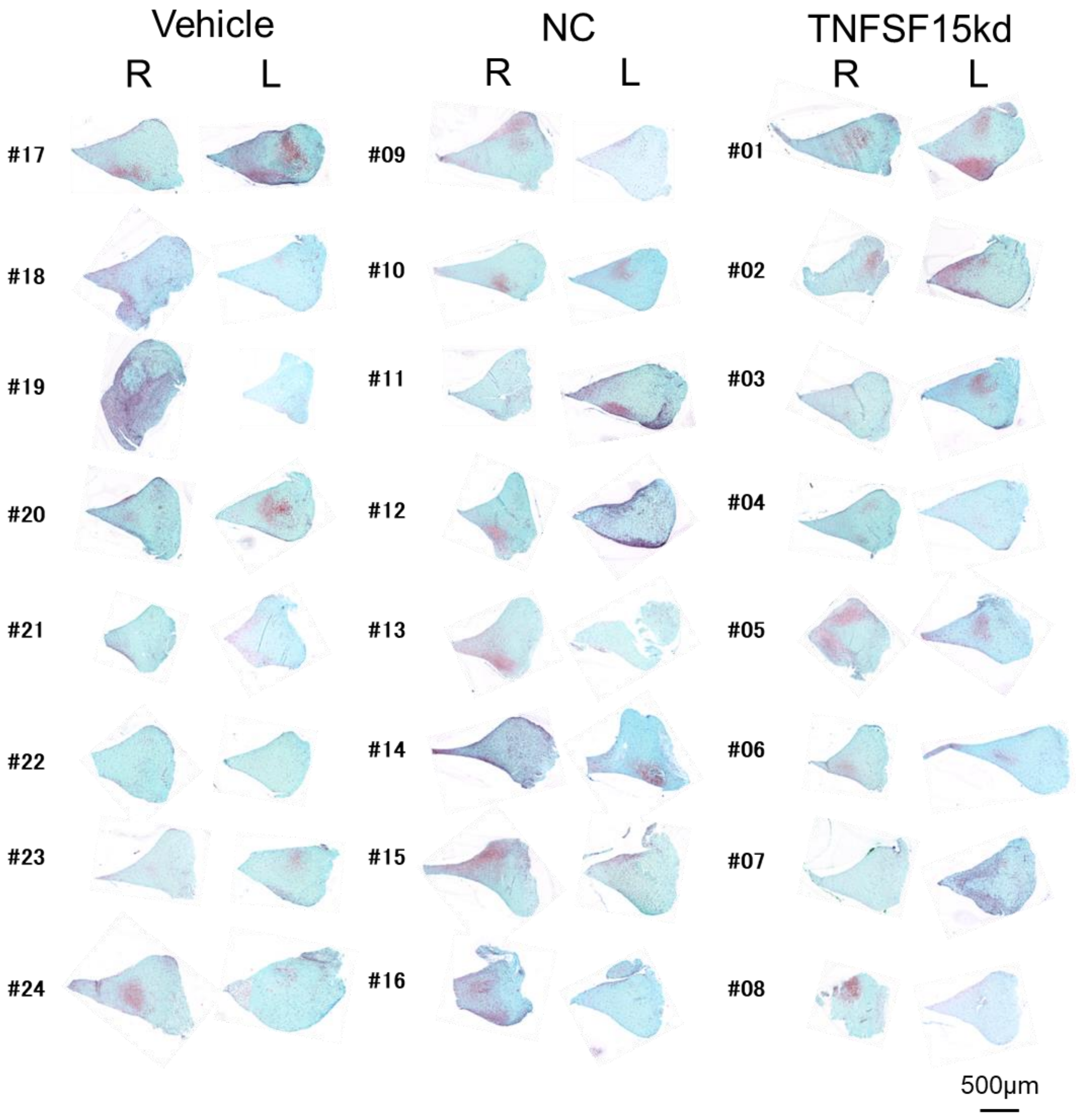

Vehicle
NC
TNFSF15kd
R
L
R
L
R
L
#17
#18
#19
#20
#21
#22
#23
#24
#09
#10
#11
#12
#13
#14
#15
#16
#01
#02
#03
#04
#05
#06
#07
#08
500μm

Supplemental Document S1. Supplemental experimental procedures

Used gene panels and databases for Amp1200 were listed here.

Qiagen,

PAHS-049Z, Osteogenesis PCR Array

PAHS-026Z, Adipogenesis PCR Array

PAHS-052Z, Innate & Adaptive Immune Response PCR Array

PAHS-011Z, Inflammatory Cytokines & Receptors PCR Array

PAHS-3803Z, Inflammatory Response and Autoimmunity 384H

PAHS-024Z, Angiogenesis PCR Array

PAHS-072Z, Angiogenic Growth Factors PCR Array

PAHS-404Z, Neurogenesis PCR Array

PAHS-031Z, Neurotrophin and Receptors PCR Array

PAHS-026Z, Fibrosis PCR Array

PAHS-128Z, Cell Motility PCR Array

PAHS-178Z, Aging PCR Array

PAHS-050Z, Cellular Senescence PCR Array

PAHS-082Z, Mesenchymal Stem Cell PCR Array

PAHS-055Z, Cell Surface Markers PCR Array

PAHS-041Z, Growth Factor PCR Array

PAHS-150Z, Cytokines and Chemokines PCR Array

Thermo Fisher Scientific,

#4414073, TaqMan™ Array Human Immune Response

EPX200-12185-901, Inflammation 20-Plex Human ProcartaPlex™ Panel

#4414071, TaqMan™ Array Human Angiogenesis

#4414094, TaqMan™ Array Human Neurotransmitters

#4418778, TaqMan™ Array, Human Extracellular Matrix & Adhesion Molecules

Illumina,

#20019169, AmpliSeq for Illumina Immune Response Panel

#20020496, AmpliSeq for Illumina RNA Inflammation Response Research Panel

BIO-RAD,

#171AL001M, Bio-Plex Pro™ Human Inflammation Panel 1, 37-Plex

#12007283, Bio-Plex Pro™ Human Cytokine Screening Panel, 48-Plex

Ontology database (http://www.informatics.jax.org/vocab/gene_ontology/)

Osteogenesis (GO:0001503), adipogenesis (GO:0045444), chondrogenesis (GO:0051216), inflammatory response (GO:0006954), angiogenesis (GO:0001525), collagen metabolic process (GO:0032963), fibroblast proliferation (GO:0048144), connective tissue replacement (GO:0097709), fibroblast migration (GO:0010761), negative regulation of cell migration (GO:0030336), positive regulation of cell migration (GO:0030335), regulation of cell migration (GO:0030334), mesenchymal stem cell migration (GO:1905319), nerve development (GO:0021675), cell adhesion mediated by integrin (GO:0033627), negative regulation of cell adhesion (GO:0007162), positive regulation of cell adhesion (GO:0045785), regulation of cell adhesion (GO:0030155), and cell aging (GO:0007569).

The 240 articles used for Amp1200 selection were referred here[1-240].

(Abdallah and Ali, 2019; Abdi et al., 2008; Ahasan et al., 2012; Alajez et al., 2018; Amable et al., 2014; Ankrum et al., 2014; Astori et al., 2007; Atasoy-Zeybek et al., 2019; Augustyniak et al., 2017; Baek et al., 2011; Baksh et al., 2004; Ball et al., 2007; Banno et al., 2016; Bartosh et al., 2010; Bellayr, 2014; Bernardo and Fibbe, 2013; Bilgen et al., 2009; Blaber et al., 2012; Boomsma and Geenen, 2012; Boxall and Jones, 2012; Burand et al., 2020; Casella et al., 2019; Chang et al., 2018; Chen et al., 2019; Chen et al., 2018; Chen et al., 2009; Churchman et al., 2017; Cieslik et al., 2014; De Bari et al., 2008;

De Becker and Riet, 2016; Deuse et al., 2011; Dexheimer et al., 2016; Diaz-Alonso et al., 2016; Djouad et al., 2007; Dong et al., 2019; E et al., 2010; EC et al., 2018; English et al., 2010; Eom et al., 2015; Eskildsen et al., 2011; Eslani et al., 2017; Fabre et al., 2019; Fan et al., 2019; Fan et al., 2020; Foppiani et al., 2019; Fracaro et al., 2020; Fu et al., 2019; Gao et al., 2016; Garcia et al., 2020; Ge et al., 2018; Ge et al., 2016; Gibson et al., 2017; Golchin et al., 2020; Gomez-Aristizabal et al., 2017; Gong et al., 2018; Guerrouahen et al., 2019; Herlofsen et al., 2013; Herlofsen et al., 2011; Hmadcha et al., 2020; Hong et al., 2020; Horita et al., 2006; Horton et al., 2013; Hostettler et al., 2017; Hsu et al., 2018; Hu et al., 2017; Hu et al., 2019; Huang et al., 2020; Huang et al., 2011; Huang et al., 2017; I et al., 2002; Idriss et al., 2018; Ip et al., 2007; Isakova et al., 2007; Islam et al., 2019a; Islam et al., 2019b; Jang et al., 2017; JH et al., 2005; Jia et al., 2019; Jiang and Xu, 2020; Joo et al., 2018; Jung et al., 2019; K and T, 2011; Kalodimou, 2016; Kanawa et al., 2018; Kanawa et al., 2019; Karagianni et al., 2013; Kehl et al., 2019; Khalilifar et al., 2019; Kim et al., 2018; Kim et al., 2012; Kokkalas et al., 2020; Kolar et al., 2017; Kolf et al., 2015; Kot et al., 2019; Kota et al., 2017; Kouroupis et al., 2019; Kulterer et al., 2007; Kuroda and Dezawa, 2014; Kurth et al., 2007; Lamichane et al., 2019; Lee et al., 2019; Lee et al., 2020; Lee et al., 2009; Lee et al., 2014; Lee et al., 2015; Leng et al., 2020; Leuning et al., 2018; Li et al., 2012; Li et al., 2014; Li et al., 2019; Li et al., 2017; Lin et al., 2017; Liu et al., 2019; Liu et al., 2006; Liu et al., 2017; Lo Surdo and Bauer, 2012; Loebel et al., 2015; Lowe et al., 2011; Luo et al., 2019; Lv et al., 2014; Lv et al., 2020; M et al., 2008; Ma et al., 2014; Maacha et al., 2020;

Madrigal et al., 2014; Mao et al., 2018; Marin-Acevedo et al., 2018; Maroni et al., 2017; Marquez-Curtis and Janowska-Wieczorek, 2013; McCully et al., 2018; McHugh and Gil, 2018; Menssen et al., 2011; Mias et al., 2009; Miceli et al., 2019; Mizukami et al., 2019; Mizuno et al., 2018; Mochizuki et al., 2006; Mueller and Tuan, 2008; Mussano et al., 2017; Mwale et al., 2006; Nakagawa et al., 2016; Nakanishi et al., 2011; Nemeth et al., 2009; Neri and Borzi, 2020; Newman et al., 2009; Nishizawa and Seki, 2016; Nitzsche et al., 2017; Noiseux et al., 2012; Oberbauer et al., 2016; Ogata et al., 2015; Oh et al., 2012; Oskowitz et al., 2011; Ozeki et al., 2016; P et al., 2006; P et al., 2011; Pardoll, 2012; Park et al., 2009; Park et al., 2019; Pittenger et al., 2019; Pleumeekers et al., 2018; Poggi and Giuliani, 2016; Pokrovskaya et al., 2020; Pokrywczynska et al., 2020; Ponte et al., 2007; Popov et al., 2011; Prockop and Oh, 2012; Qi et al., 2016; Qiong et al., 2020; Rahbarghazi, 2019; Rakic et al., 2018; Ratushnyy et al., 2020; Reinders and Hoogduijn, 2014; Ren et al., 2008; Ren et al., 2010; Rengasamy et al., 2017; Ribeiro et al., 2019; Riekstina et al., 2009; RK et al., 2000; Roddy et al., 2011; Rogers et al., 2020; Romieu-Mourez et al., 2009; Rossi et al., 2019; Sakaguchi et al., 2005; Salama et al., 2014; Sanchez et al., 2017; Sane et al., 2018; Sasaki et al., 2009; Schallmoser et al., 2010; Schutze et al., 2005; Segers et al., 2006; Sekiya et al., 2012; Shibata et al., 2007; Sofia et al., 2019; Sohni and Verfaillie, 2013; Somaiah et al., 2015; Song et al., 2020; Sordi et al., 2005; Spees et al., 2016; Studle et al., 2019; Sun et al., 2020; Sung et al., 2015; Tao et al., 2016; Tebebi et al., 2017; Teixeira et al., 2017; Terunuma et al., 2019; Tofino-Vian et al., 2018; Tsai et al., 2009; Tucker et al., 2020; Turinetto

et al., 2016; Ullah et al., 2019; Usunier et al., 2014; van Buul et al., 2012; Vanden Berg-Foels, 2014; W et al., 2010; Wagner et al., 2008; Wang et al., 2019; Wang et al., 2018; Watt et al., 2013; Weiss and Dahlke, 2019; Weiss et al., 2006; Wiese et al., 2019; Wu et al., 2018; Wu et al., 2019; Wykes and Lewin, 2018; Xu et al., 2018; Xu et al., 2008; YA et al., 2005; Yagi et al., 2010; Yang et al., 2011; Yang et al., 2018; Yao et al., 2016; Yoshida et al., 2018; Yu and Kang, 2013; Yu et al., 2016; Z and HW, 2019; Zhang et al., 2018; Zhang et al., 2013; Zhou et al., 2008; Zhu et al., 2020; Zwolanek et al., 2015)

Supplemental references for the Amp1200 selection

1. Abdallah, B.M., and Ali, E.M. (2019). 5'-hydroxy Auraptene stimulates osteoblast differentiation of bone marrow-derived mesenchymal stem cells via a BMP-dependent mechanism. J Biomed Sci *26*, 51. 10.1186/s12929-019-0544-7.
2. Abdi, R., Fiorina, P., Adra, C.N., Atkinson, M., and Sayegh, M.H. (2008). Immunomodulation by mesenchymal stem cells: a potential therapeutic strategy for type 1 diabetes. Diabetes *57*, 1759-1767. 10.2337/db08-0180.
3. Ahasan, M.M., Hardy, R., Jones, C., Kaur, K., Nanus, D., Juarez, M., Morgan, S.A., Hassan-Smith, Z., Benezech, C., Caamano, J.H., et al. (2012). Inflammatory regulation of glucocorticoid metabolism in mesenchymal stromal cells. Arthritis Rheum *64*, 2404-2413. 10.1002/art.34414.
4. Alajez, N.M., Al-Ali, D., Vishnubalaji, R., Manikandan, M., Alfayez, M., Kassem, M., and Aldahmash,

A. (2018). Which stem cells to choose for regenerative medicine application Bone marrow and adipose tissue stromal stem cells. Journal of Nature and Science of Medicine *1(2)*, 48-54. 10.4103/JNSM.JNSM_18_18.

5. Amable, P.R., Teixeira, M.V., Carias, R.B., Granjeiro, J.M., and Borojevic, R. (2014). Protein synthesis and secretion in human mesenchymal cells derived from bone marrow, adipose tissue and Wharton's jelly. Stem Cell Res Ther *5*, 53. 10.1186/scrt442.

6. Ankrum, J.A., Dastidar, R.G., Ong, J.F., Levy, O., and Karp, J.M. (2014). Performance-enhanced mesenchymal stem cells via intracellular delivery of steroids. Sci Rep *4*, 4645. 10.1038/srep04645.

7. Astori, G., Vignati, F., Bardelli, S., Tubio, M., Gola, M., Albertini, V., Bambi, F., Scali, G., Castelli, D., Rasini, V., et al. (2007). "In vitro" and multicolor phenotypic characterization of cell subpopulations identified in fresh human adipose tissue stromal vascular fraction and in the derived mesenchymal stem cells. J Transl Med *5*, 55. 10.1186/1479-5876-5-55.

8. Atasoy-Zeybek, A., Ivkovic, A., Beyzadeoglu, T., Onal, A., Evans, C.H., and Kose, G.T. (2019). Paracrine effects of living human bone particles on the osteogenic differentiation of mesenchymal stem cells. Eur Cell Mater *38*, 14-22. 10.22203/eCM.v038a02.

9. Augustyniak, E., Suchorska, W.M., Trzeciak, T., and Richter, M. (2017). Gene expression profile in human induced pluripotent stem cells: Chondrogenic differentiation in vitro, part B. Mol Med Rep *15*, 2402-2414. 10.3892/mmr.2017.6335.

10. Baek, S.J., Kang, S.K., and Ra, J.C. (2011). In vitro migration capacity of human adipose tissue-derived mesenchymal stem cells reflects their expression of receptors for chemokines and growth factors. Exp Mol Med *43*, 596-603. 10.3858/emm.2011.43.10.069.

11. Baksh, D., Song, L., and Tuan, R.S. (2004). Adult mesenchymal stem cells: characterization, differentiation, and application in cell and gene therapy. J Cell Mol Med *8*, 301-316. 10.1111/j.1582-4934.2004.tb00320.x.

12. Ball, S.G., Shuttleworth, C.A., and Kielty, C.M. (2007). Vascular endothelial growth factor can signal through platelet-derived growth factor receptors. J Cell Biol *177*, 489-500. 10.1083/jcb.200608093.

13. Banno, K., Omori, S., Hirata, K., Nawa, N., Nakagawa, N., Nishimura, K., Ohtaka, M., Nakanishi, M., Sakuma, T., Yamamoto, T., et al. (2016). Systematic Cellular Disease Models Reveal Synergistic Interaction of Trisomy 21 and GATA1 Mutations in Hematopoietic Abnormalities. Cell Rep *15*, 1228-1241. 10.1016/j.celrep.2016.04.031.

14. Bartosh, T.J., Ylostalo, J.H., Mohammadipoor, A., Bazhanov, N., Coble, K., Claypool, K., Lee, R.H., Choi, H., and Prockop, D.J. (2010). Aggregation of human mesenchymal stromal cells (MSCs) into 3D spheroids enhances their antiinflammatory properties. Proc Natl Acad Sci U S A *107*, 13724-13729. 10.1073/pnas.1008117107.

15. Bellayr, I.H.C., Jennifer G;Lababidi, Samir;Yang, Amy X;Lo Surdo, Jessica L;Bauer, Steven R;Puri, Raj K (2014). Gene markers of cellular aging in human multipotent stromal cells in culture. Stem Cell

Research & Therapy *5*, 1-22. doi:10.1186/scrt448.

16. Bernardo, M.E., and Fibbe, W.E. (2013). Mesenchymal stromal cells: sensors and switchers of inflammation. Cell Stem Cell *13*, 392-402. 10.1016/j.stem.2013.09.006.

17. Bilgen, B., Ren, Y., Pei, M., Aaron, R.K., and Ciombor, D.M. (2009). CD14-negative isolation enhances chondrogenesis in synovial fibroblasts. Tissue Eng Part A *15*, 3261-3270. 10.1089/ten.TEA.2008.0273.

18. Blaber, S.P., Webster, R.A., Hill, C.J., Breen, E.J., Kuah, D., Vesey, G., and Herbert, B.R. (2012). Analysis of in vitro secretion profiles from adipose-derived cell populations. J Transl Med *10*, 172. 10.1186/1479-5876-10-172.

19. Boomsma, R.A., and Geenen, D.L. (2012). Mesenchymal stem cells secrete multiple cytokines that promote angiogenesis and have contrasting effects on chemotaxis and apoptosis. PLoS One *7*, e35685. 10.1371/journal.pone.0035685.

20. Boxall, S.A., and Jones, E. (2012). Markers for characterization of bone marrow multipotential stromal cells. Stem Cells Int *2012*, 975871. 10.1155/2012/975871.

21. Burand, A.J., Jr., Di, L., Boland, L.K., Boyt, D.T., Schrodt, M.V., Santillan, D.A., and Ankrum, J.A. (2020). Aggregation of Human Mesenchymal Stromal Cells Eliminates Their Ability to Suppress Human T Cells. Front Immunol *11*, 143. 10.3389/fimmu.2020.00143.

22. Casella, G., Munk, R., Kim, K.M., Piao, Y., De, S., Abdelmohsen, K., and Gorospe, M. (2019).

Transcriptome signature of cellular senescence. Nucleic Acids Res *47*, 7294-7305. 10.1093/nar/gkz555.

23. Chang, P., Zhang, B., Shao, L., Song, W., Shi, W., Wang, L., Xu, T., Li, D., Gao, X., Qu, Y., et al. (2018). Mesenchymal stem cells over-expressing cxcl12 enhance the radioresistance of the small intestine. Cell Death Dis *9*, 154. 10.1038/s41419-017-0222-1.

24. Chen, G., Zhong, L., Wang, Q., Li, Z., Shang, J., Yang, Q., Du, Z., Wang, J., Song, Y., and Zhang, G. (2019). The expression of chondrogenesis-related and arthritis-related genes in human ONFH cartilage with different Ficat stages. PeerJ *7*, e6306. 10.7717/peerj.6306.

25. Chen, M.S., Lin, C.Y., Chiu, Y.H., Chen, C.P., Tsai, P.J., and Wang, H.S. (2018). IL-1beta-Induced Matrix Metalloprotease-1 Promotes Mesenchymal Stem Cell Migration via PAR1 and G-Protein-Coupled Signaling Pathway. Stem Cells Int *2018*, 3524759. 10.1155/2018/3524759.

26. Chen, W.H., Lai, M.T., Wu, A.T.H., Wu, C.C., Gelovani, J.G., Lin, C.T., Hung, S.C., Chiu, W.T., and Deng, W.P. (2009). In vitro stage-specific chondrogenesis of mesenchymal stem cells committed to chondrocytes. Arthritis & Rheumatism *60*, 450-459. 10.1002/art.24265.

27. Churchman, S.M., Boxall, S.A., McGonagle, D., and Jones, E.A. (2017). Predicting the Remaining Lifespan and Cultivation-Related Loss of Osteogenic Capacity of Bone Marrow Multipotential Stromal Cells Applicable across a Broad Donor Age Range. Stem Cells Int *2017*, 6129596. 10.1155/2017/6129596.

28. Cieslik, K.A., Trial, J., Crawford, J.R., Taffet, G.E., and Entman, M.L. (2014). Adverse fibrosis in the aging heart depends on signaling between myeloid and mesenchymal cells; role of inflammatory fibroblasts. J Mol Cell Cardiol *70*, 56-63. 10.1016/j.yjmcc.2013.10.017.

29. De Bari, C., Dell'Accio, F., Karystinou, A., Guillot, P.V., Fisk, N.M., Jones, E.A., McGonagle, D., Khan, I.M., Archer, C.W., Mitsiadis, T.A., et al. (2008). A biomarker-based mathematical model to predict bone-forming potency of human synovial and periosteal mesenchymal stem cells. Arthritis Rheum *58*, 240-250. 10.1002/art.23143.

30. De Becker, A., and Riet, I.V. (2016). Homing and migration of mesenchymal stromal cells: How to improve the efficacy of cell therapy? World J Stem Cells *8*, 73-87. 10.4252/wjsc.v8.i3.73.

31. Deuse, T., Stubbendorff, M., Tang-Quan, K., Phillips, N., Kay, M.A., Eiermann, T., Phan, T.T., Volk, H.D., Reichenspurner, H., Robbins, R.C., and Schrepfer, S. (2011). Immunogenicity and immunomodulatory properties of umbilical cord lining mesenchymal stem cells. Cell Transplant *20*, 655-667. 10.3727/096368910X536473.

32. Dexheimer, V., Gabler, J., Bomans, K., Sims, T., Omlor, G., and Richter, W. (2016). Differential expression of TGF-beta superfamily members and role of Smad1/5/9-signalling in chondral versus endochondral chondrocyte differentiation. Sci Rep *6*, 36655. 10.1038/srep36655.

33. Diaz-Alonso, J., Paraiso-Luna, J., Navarrete, C., Del Rio, C., Cantarero, I., Palomares, B., Aguareles, J., Fernandez-Ruiz, J., Bellido, M.L., Pollastro, F., et al. (2016). VCE-003.2, a novel cannabigerol

derivative, enhances neuronal progenitor cell survival and alleviates symptomatology in murine models of Huntington's disease. Sci Rep *6*, 29789. 10.1038/srep29789.

34. Djouad, F., Delorme, B., Maurice, M., Bony, C., Apparailly, F., Louis-Plence, P., Canovas, F., Charbord, P., Noel, D., and Jorgensen, C. (2007). Microenvironmental changes during differentiation of mesenchymal stem cells towards chondrocytes. Arthritis Res Ther *9*, R33. 10.1186/ar2153.

35. Dong, R., Liu, Y., Yang, Y., Wang, H., Xu, Y., and Zhang, Z. (2019). MSC-Derived Exosomes-Based Therapy for Peripheral Nerve Injury: A Novel Therapeutic Strategy. Biomed Res Int *2019*, 6458237. 10.1155/2019/6458237.

36. E, J., SM, C., A, E., MH, B., EA, H., CH, B., R, R., S, K., P, E., D, M., and F, P. (2010). Mesenchymal stem cells in rheumatoid synovium: enumeration and functional assessment in relation to synovial inflammation level. Annals of the rheumatic diseases *69*. 10.1136/ard.2008.106435.

37. EC, B., HS, W., J, F., S, K., K, H., and M, O. (2018). Independent chondrogenic potential of canine bone marrow-derived mesenchymal stem cells in monolayer expansion cultures decreases in a passage-dependent pattern. The Journal of veterinary medical science *80*. 10.1292/jvms.18-0202.

38. English, K., French, A., and Wood, K.J. (2010). Mesenchymal stromal cells: facilitators of successful transplantation? Cell Stem Cell *7*, 431-442. 10.1016/j.stem.2010.09.009.

39. Eom, Y.W., Shim, K.Y., and Baik, S.K. (2015). Mesenchymal stem cell therapy for liver fibrosis. Korean J Intern Med *30*, 580-589. 10.3904/kjim.2015.30.5.580.

40. Eskildsen, T., Taipaleenmaki, H., Stenvang, J., Abdallah, B.M., Ditzel, N., Nossent, A.Y., Bak, M., Kauppinen, S., and Kassem, M. (2011). MicroRNA-138 regulates osteogenic differentiation of human stromal (mesenchymal) stem cells in vivo. Proc Natl Acad Sci U S A *108*, 6139-6144. 10.1073/pnas.1016758108.

41. Eslani, M., Putra, I., Shen, X., Hamouie, J., Afsharkhamseh, N., Besharat, S., Rosenblatt, M.I., Dana, R., Hematti, P., and Djalilian, A.R. (2017). Corneal Mesenchymal Stromal Cells Are Directly Antiangiogenic via PEDF and sFLT-1. Invest Ophthalmol Vis Sci *58*, 5507-5517. 10.1167/iovs.17-22680.

42. Fabre, H., Ducret, M., Degoul, O., Rodriguez, J., Perrier-Groult, E., Aubert-Foucher, E., Pasdeloup, M., Auxenfans, C., McGuckin, C., Forraz, N., and Mallein-Gerin, F. (2019). Characterization of Different Sources of Human MSCs Expanded in Serum-Free Conditions with Quantification of Chondrogenic Induction in 3D. Stem Cells Int *2019*, 2186728. 10.1155/2019/2186728.

43. Fan, F.Y., Deng, R., Lai, S.H., Wen, Q., Zeng, Y., Gao, L., Liu, Y., Kong, P., Zhong, J., Su, Y., and Zhang, X. (2019). Inhibition of microRNA-221-5p induces osteogenic differentiation by directly targeting smad3 in myeloma bone disease mesenchymal stem cells. Oncol Lett *18*, 6536-6544. 10.3892/ol.2019.10992.

44. Fan, X.L., Zhang, Y., Li, X., and Fu, Q.L. (2020). Mechanisms underlying the protective effects of mesenchymal stem cell-based therapy. Cell Mol Life Sci *77*, 2771-2794. 10.1007/s00018-020-03454-

6.

45. Foppiani, E.M., Candini, O., Mastrolia, I., Murgia, A., Grisendi, G., Samarelli, A.V., Boscaini, G., Pacchioni, L., Pinelli, M., De Santis, G., et al. (2019). Impact of HOXB7 overexpression on human adipose-derived mesenchymal progenitors. Stem Cell Res Ther *10*, 101. 10.1186/s13287-019-1200-6.

46. Fracaro, L., Senegaglia, A.C., Herai, R.H., Leitolis, A., Boldrini-Leite, L.M., Rebelatto, C.L.K., Travers, P.J., Brofman, P.R.S., and Correa, A. (2020). The Expression Profile of Dental Pulp-Derived Stromal Cells Supports Their Limited Capacity to Differentiate into Adipogenic Cells. Int J Mol Sci *21*. 10.3390/ijms21082753.

47. Fu, X., Liu, G., Halim, A., Ju, Y., Luo, Q., and Song, A.G. (2019). Mesenchymal Stem Cell Migration and Tissue Repair. Cells *8*. 10.3390/cells8080784.

48. Gao, F., Chiu, S.M., Motan, D.A., Zhang, Z., Chen, L., Ji, H.L., Tse, H.F., Fu, Q.L., and Lian, Q. (2016). Mesenchymal stem cells and immunomodulation: current status and future prospects. Cell Death Dis *7*, e2062. 10.1038/cddis.2015.327.

49. Garcia, J., Hulme, C., Mennan, C., Roberts, S., Bastiaansen-Jenniskens, Y.M., van Osch, G., Tins, B., Gallacher, P., and Wright, K. (2020). The synovial fluid from patients with focal cartilage defects contains mesenchymal stem/stromal cells and macrophages with pro- and anti-inflammatory phenotypes. Osteoarthr Cartil Open *2*, 100039. 10.1016/j.ocarto.2020.100039.

50. Ge, J., Burnier, L., Adamopoulou, M., Kwa, M.Q., Schaks, M., Rottner, K., and Brakebusch, C. (2018). RhoA, Rac1, and Cdc42 differentially regulate alphaSMA and collagen I expression in mesenchymal stem cells. J Biol Chem *293*, 9358-9369. 10.1074/jbc.RA117.001113.

51. Ge, L., Jiang, M., Duan, D., Wang, Z., Qi, L., Teng, X., Zhao, Z., Wang, L., Zhuo, Y., Chen, P., et al. (2016). Secretome of Olfactory Mucosa Mesenchymal Stem Cell, a Multiple Potential Stem Cell. Stem Cells Int *2016*, 1243659. 10.1155/2016/1243659.

52. Gibson, J.D., O'Sullivan, M.B., Alaee, F., Paglia, D.N., Yoshida, R., Guzzo, R.M., and Drissi, H. (2017). Regeneration of Articular Cartilage by Human ESC-Derived Mesenchymal Progenitors Treated Sequentially with BMP-2 and Wnt5a. Stem Cells Transl Med *6*, 40-50. 10.5966/sctm.2016-0020.

53. Golchin, A., Seyedjafari, E., and Ardeshirylajimi, A. (2020). Mesenchymal Stem Cell Therapy for COVID-19: Present or Future. Stem Cell Rev Rep *16*, 427-433. 10.1007/s12015-020-09973-w.

54. Gomez-Aristizabal, A., Sharma, A., Bakooshli, M.A., Kapoor, M., Gilbert, P.M., Viswanathan, S., and Gandhi, R. (2017). Stage-specific differences in secretory profile of mesenchymal stromal cells (MSCs) subjected to early- vs late-stage OA synovial fluid. Osteoarthritis Cartilage *25*, 737-741. 10.1016/j.joca.2016.11.010.

55. Gong, M., Liang, T., Zhang, H., Chen, S., Hu, Y., Zhou, J., Zhang, X., Zhang, W., Geng, X., and Zou, X. (2018). Gene expression profiling: identification of gene expression in human MSC chondrogenic

differentiation. Am J Transl Res *10*, 3555-3566.

56. Guerrouahen, B.S., Sidahmed, H., Al Sulaiti, A., Al Khulaifi, M., and Cugno, C. (2019). Enhancing Mesenchymal Stromal Cell Immunomodulation for Treating Conditions Influenced by the Immune System. Stem Cells Int *2019*, 7219297. 10.1155/2019/7219297.

57. Herlofsen, S.R., Bryne, J.C., Hoiby, T., Wang, L., Issner, R., Zhang, X., Coyne, M.J., Boyle, P., Gu, H., Meza-Zepeda, L.A., et al. (2013). Genome-wide map of quantified epigenetic changes during in vitro chondrogenic differentiation of primary human mesenchymal stem cells. BMC Genomics *14*, 105. 10.1186/1471-2164-14-105.

58. Herlofsen, S.R., Kuchler, A.M., Melvik, J.E., and Brinchmann, J.E. (2011). Chondrogenic differentiation of human bone marrow-derived mesenchymal stem cells in self-gelling alginate discs reveals novel chondrogenic signature gene clusters. Tissue Eng Part A *17*, 1003-1013. 10.1089/ten.TEA.2010.0499.

59. Hmadcha, A., Martin-Montalvo, A., Gauthier, B.R., Soria, B., and Capilla-Gonzalez, V. (2020). Therapeutic Potential of Mesenchymal Stem Cells for Cancer Therapy. Front Bioeng Biotechnol *8*, 43. 10.3389/fbioe.2020.00043.

60. Hong, Y., He, H., Jiang, G., Zhang, H., Tao, W., Ding, Y., Yuan, D., Liu, J., Fan, H., Lin, F., et al. (2020). miR-155-5p inhibition rejuvenates aged mesenchymal stem cells and enhances cardioprotection following infarction. Aging Cell *19*, e13128. 10.1111/acel.13128.

61. Horita, Y., Honmou, O., Harada, K., Houkin, K., Hamada, H., and Kocsis, J.D. (2006). Intravenous administration of glial cell line-derived neurotrophic factor gene-modified human mesenchymal stem cells protects against injury in a cerebral ischemia model in the adult rat. J Neurosci Res *84*, 1495-1504. 10.1002/jnr.21056.

62. Horton, J.A., Hudak, K.E., Chung, E.J., White, A.O., Scroggins, B.T., Burkeen, J.F., and Citrin, D.E. (2013). Mesenchymal stem cells inhibit cutaneous radiation-induced fibrosis by suppressing chronic inflammation. Stem Cells *31*, 2231-2241. 10.1002/stem.1483.

63. Hostettler, K.E., Gazdhar, A., Khan, P., Savic, S., Tamo, L., Lardinois, D., Roth, M., Tamm, M., and Geiser, T. (2017). Multipotent mesenchymal stem cells in lung fibrosis. PLoS One *12*, e0181946. 10.1371/journal.pone.0181946.

64. Hsu, M.F., Yu, S.H., Chuang, S.J., Kuo, T.K., Singal, P.K., Huang, C.Y., Kao, C.L., and Kuo, C.H. (2018). Can mesenchymal stem cell lysate reverse aging? Aging (Albany NY) *10*, 2900-2910. 10.18632/aging.101595.

65. Hu, K., Sun, H., Gui, B., and Sui, C. (2017). Gremlin-1 suppression increases BMP-2-induced osteogenesis of human mesenchymal stem cells. Mol Med Rep *15*, 2186-2194. 10.3892/mmr.2017.6253.

66. Hu, N., Gao, Y., Jayasuriya, C.T., Liu, W., Du, H., Ding, J., Feng, M., and Chen, Q. (2019). Chondrogenic induction of human osteoarthritic cartilage-derived mesenchymal stem cells activates

mineralization and hypertrophic and osteogenic gene expression through a mechanomiR. Arthritis Res Ther *21*, 167. 10.1186/s13075-019-1949-0.

67. Huang, P., Li, Y., Xu, C., Melino, G., Shao, C., and Shi, Y. (2020). HSD11B1 is upregulated synergistically by IFNgamma and TNFalpha and mediates TSG-6 expression in human UC-MSCs. Cell Death Discov *6*, 24. 10.1038/s41420-020-0262-7.

68. Huang, P.I., Chen, Y.C., Chen, L.H., Juan, C.C., Ku, H.H., Wang, S.T., Chiou, S.H., Chiou, G.Y., Chi, C.W., Hsu, C.C., et al. (2011). PGC-1alpha mediates differentiation of mesenchymal stem cells to brown adipose cells. J Atheroscler Thromb *18*, 966-980. 10.5551/jat.7401.

69. Huang, S., Song, X., Li, T., Xiao, J., Chen, Y., Gong, X., Zeng, W., Yang, L., and Chen, C. (2017). Pellet coculture of osteoarthritic chondrocytes and infrapatellar fat pad-derived mesenchymal stem cells with chitosan/hyaluronic acid nanoparticles promotes chondrogenic differentiation. Stem Cell Res Ther *8*, 264. 10.1186/s13287-017-0719-7.

70. I, S., JT, V., BL, L., and DJ, P. (2002). In vitro cartilage formation by human adult stem cells from bone marrow stroma defines the sequence of cellular and molecular events during chondrogenesis. Proceedings of the National Academy of Sciences of the United States of America *99*. 10.1073/pnas.052716199.

71. Idriss, N.K., Sayyed, H.G., Osama, A., and Sabry, D. (2018). Treatment Efficiency of Different Routes of Bone Marrow-Derived Mesenchymal Stem Cell Injection in Rat Liver Fibrosis Model. Cell Physiol

Biochem *48*, 2161-2171. 10.1159/000492558.

72. Ip, J.E., Wu, Y., Huang, J., Zhang, L., Pratt, R.E., and Dzau, V.J. (2007). Mesenchymal stem cells use integrin beta1 not CXC chemokine receptor 4 for myocardial migration and engraftment. Mol Biol Cell *18*, 2873-2882. 10.1091/mbc.e07-02-0166.

73. Isakova, I.A., Baker, K., DuTreil, M., Dufour, J., Gaupp, D., and Phinney, D.G. (2007). Age- and dose-related effects on MSC engraftment levels and anatomical distribution in the central nervous systems of nonhuman primates: identification of novel MSC subpopulations that respond to guidance cues in brain. Stem Cells *25*, 3261-3270. 10.1634/stemcells.2007-0543.

74. Islam, A., Fossum, V., Hansen, A.K., Urbarova, I., Knutsen, G., and Martinez-Zubiaurre, I. (2019a). In vitro chondrogenic potency of surplus chondrocytes from autologous transplantation procedures does not predict short-term clinical outcomes. BMC Musculoskelet Disord *20*, 19. 10.1186/s12891-018-2380-4.

75. Islam, A., Urbarova, I., Bruun, J.A., and Martinez-Zubiaurre, I. (2019b). Large-scale secretome analyses unveil the superior immunosuppressive phenotype of umbilical cord stromal cells as compared to other adult mesenchymal stromal cells. Eur Cell Mater *37*, 153-174. 10.22203/eCM.v037a10.

76. Jang, Y.J., An, S.Y., and Kim, J.H. (2017). Identification of MFGE8 in mesenchymal stem cell secretome as an anti-fibrotic factor in liver fibrosis. BMB Rep *50*, 58-59.

10.5483/bmbrep.2017.50.2.012.

77. JH, H., ES, H., MT, M., A, A., Y, T., R, K., E, M., T, B., BM, S., PA, S., et al. (2005). TAZ, a transcriptional modulator of mesenchymal stem cell differentiation. Science (New York, N.Y.) *309*. 10.1126/science.1110955.

78. Jia, Z., Wang, S., Liang, Y., and Liu, Q. (2019). Combination of kartogenin and transforming growth factor-beta3 supports synovial fluid-derived mesenchymal stem cell-based cartilage regeneration. Am J Transl Res *11*, 2056-2069.

79. Jiang, W., and Xu, J. (2020). Immune modulation by mesenchymal stem cells. Cell Prolif *53*, e12712. 10.1111/cpr.12712.

80. Joo, K.H., Song, J.S., Kim, S., Lee, H.S., Jeon, M., Kim, S.O., and Lee, J.H. (2018). Cytokine Expression of Stem Cells Originating from the Apical Complex and Coronal Pulp of Immature Teeth. J Endod *44*, 87-92 e81. 10.1016/j.joen.2017.08.018.

81. Jung, J.S., Volk, C., Marga, C., Navarrete Santos, A., Jung, M., Rujescu, D., and Navarrete Santos, A. (2019). Adipose-Derived Stem/Stromal Cells Recapitulate Aging Biomarkers and Show Reduced Stem Cell Plasticity Affecting Their Adipogenic Differentiation Capacity. Cell Reprogram *21*, 187-199. 10.1089/cell.2019.0010.

82. K, J., and T, N. (2011). Human dermal fibroblasts exhibit delayed adipogenic differentiation compared with mesenchymal stem cells. Stem cells and development *20*. 10.1089/scd.2010.0258.

83. Kalodimou, V.E. (2016). Mesenchymal Stem Cells Markers. Open Access J Neurol Neurosurg *1(3)*, 555561.DOI. http://dx.doi.org/10.19080/OAJNN.2016.01.555561.

84. Kanawa, M., Igarashi, A., Fujimoto, K., Higashi, Y., Kurihara, H., Sugiyama, M., Saskianti, T., Kato, Y., and Kawamoto, T. (2018). Genetic Markers Can Predict Chondrogenic Differentiation Potential in Bone Marrow-Derived Mesenchymal Stromal Cells. Stem Cells Int *2018*, 9530932. 10.1155/2018/9530932.

85. Kanawa, M., Igarashi, A., Fujimoto, K., Ronald, V.S., Higashi, Y., Kurihara, H., Kato, Y., and Kawamoto, T. (2019). Potential Marker Genes for Predicting Adipogenic Differentiation of Mesenchymal Stromal Cells. Applied Sciences *9*. 10.3390/app9142942.

86. Karagianni, M., Brinkmann, I., Kinzebach, S., Grassl, M., Weiss, C., Bugert, P., and Bieback, K. (2013). A comparative analysis of the adipogenic potential in human mesenchymal stromal cells from cord blood and other sources. Cytotherapy *15*, 76-88. 10.1016/j.jcyt.2012.11.001.

87. Kehl, D., Generali, M., Mallone, A., Heller, M., Uldry, A.C., Cheng, P., Gantenbein, B., Hoerstrup, S.P., and Weber, B. (2019). Proteomic analysis of human mesenchymal stromal cell secretomes: a systematic comparison of the angiogenic potential. NPJ Regen Med *4*, 8. 10.1038/s41536-019-0070-y.

88. Khalilifar, M.A., Baghaban Eslaminejad, M.R., Ghasemzadeh, M., Hosseini, S., and Baharvand, H. (2019). In Vitro and In Vivo Comparison of Different Types of Rabbit Mesenchymal Stem Cells for

Cartilage Repair. Cell J *21*, 150-160. 10.22074/cellj.2019.6149.

89. Kim, D.H., Lim, H., Lee, D., Choi, S.J., Oh, W., Yang, Y.S., Oh, J.S., Hwang, H.H., and Jeon, H.B. (2018). Thrombospondin-1 secreted by human umbilical cord blood-derived mesenchymal stem cells rescues neurons from synaptic dysfunction in Alzheimer's disease model. Sci Rep *8*, 354. 10.1038/s41598-017-18542-0.

90. Kim, J.Y., Kim, D.H., Kim, J.H., Lee, D., Jeon, H.B., Kwon, S.J., Kim, S.M., Yoo, Y.J., Lee, E.H., Choi, S.J., et al. (2012). Soluble intracellular adhesion molecule-1 secreted by human umbilical cord blood-derived mesenchymal stem cell reduces amyloid-beta plaques. Cell Death Differ *19*, 680-691. 10.1038/cdd.2011.140.

91. Kokkalas, N., Kokotis, P., Diamantopoulou, K., Galanos, A., Lelovas, P., Papachristou, D.J., Dontas, I.A., and Triantafyllopoulos, I.K. (2020). Platelet-rich Plasma and Mesenchymal Stem Cells Local Infiltration Promote Functional Recovery and Histological Repair of Experimentally Transected Sciatic Nerves in Rats. Cureus *12*, e8262. 10.7759/cureus.8262.

92. Kolar, M.K., Itte, V.N., Kingham, P.J., Novikov, L.N., Wiberg, M., and Kelk, P. (2017). The neurotrophic effects of different human dental mesenchymal stem cells. Sci Rep *7*, 12605. 10.1038/s41598-017-12969-1.

93. Kolf, C.M., Song, L., Helm, J., and Tuan, R.S. (2015). Nascent osteoblast matrix inhibits osteogenesis of human mesenchymal stem cells in vitro. Stem Cell Res Ther *6*, 258. 10.1186/s13287-015-0223-x.

94. Kot, M., Musial-Wysocka, A., Lasota, M., Ulman, A., and Majka, M. (2019). Secretion, migration and adhesion as key processes in the therapeutic activity of mesenchymal stem cells. Acta Biochim Pol *66*, 499-507. 10.18388/abp.2019_2895.

95. Kota, D.J., Prabhakara, K.S., Toledano-Furman, N., Bhattarai, D., Chen, Q., DiCarlo, B., Smith, P., Triolo, F., Wenzel, P.L., Cox, C.S., Jr., and Olson, S.D. (2017). Prostaglandin E2 Indicates Therapeutic Efficacy of Mesenchymal Stem Cells in Experimental Traumatic Brain Injury. Stem Cells *35*, 1416-1430. 10.1002/stem.2603.

96. Kouroupis, D., Bowles, A.C., Willman, M.A., Perucca Orfei, C., Colombini, A., Best, T.M., Kaplan, L.D., and Correa, D. (2019). Infrapatellar fat pad-derived MSC response to inflammation and fibrosis induces an immunomodulatory phenotype involving CD10-mediated Substance P degradation. Sci Rep *9*, 10864. 10.1038/s41598-019-47391-2.

97. Kulterer, B., Friedl, G., Jandrositz, A., Sanchez-Cabo, F., Prokesch, A., Paar, C., Scheideler, M., Windhager, R., Preisegger, K.H., and Trajanoski, Z. (2007). Gene expression profiling of human mesenchymal stem cells derived from bone marrow during expansion and osteoblast differentiation. BMC Genomics *8*, 70. 10.1186/1471-2164-8-70.

98. Kuroda, Y., and Dezawa, M. (2014). Mesenchymal stem cells and their subpopulation, pluripotent muse cells, in basic research and regenerative medicine. Anat Rec (Hoboken) *297*, 98-110. 10.1002/ar.22798.

99. Kurth, T., Hedbom, E., Shintani, N., Sugimoto, M., Chen, F.H., Haspl, M., Martinovic, S., and Hunziker, E.B. (2007). Chondrogenic potential of human synovial mesenchymal stem cells in alginate. Osteoarthritis Cartilage *15*, 1178-1189. 10.1016/j.joca.2007.03.015.

100. Lamichane, S., Baek, S.H., Kim, Y.J., Park, J.H., Dahal Lamichane, B., Jang, W.B., Ji, S., Lee, N.K., Dehua, L., Kim, D.Y., et al. (2019). MHY2233 Attenuates Replicative Cellular Senescence in Human Endothelial Progenitor Cells via SIRT1 Signaling. Oxid Med Cell Longev *2019*, 6492029. 10.1155/2019/6492029.

101. Lee, H.J., Choi, B., Kim, Y., Lee, S.E., Jin, H.J., Lee, H.S., Chang, E.J., and Kim, S.W. (2019). The Upregulation of Toll-Like Receptor 3 via Autocrine IFN-beta Signaling Drives the Senescence of Human Umbilical Cord Blood-Derived Mesenchymal Stem Cells Through JAK1. Front Immunol *10*, 1659. 10.3389/fimmu.2019.01659.

102. Lee, H.J., Ko, J.H., Kim, H.J., Jeong, H.J., and Oh, J.Y. (2020). Mesenchymal stromal cells induce distinct myeloid-derived suppressor cells in inflammation. JCI Insight *5*. 10.1172/jci.insight.136059.

103. Lee, R.H., Pulin, A.A., Seo, M.J., Kota, D.J., Ylostalo, J., Larson, B.L., Semprun-Prieto, L., Delafontaine, P., and Prockop, D.J. (2009). Intravenous hMSCs improve myocardial infarction in mice because cells embolized in lung are activated to secrete the anti-inflammatory protein TSG-6. Cell Stem Cell *5*, 54-63. 10.1016/j.stem.2009.05.003.

104. Lee, R.H., Yu, J.M., Foskett, A.M., Peltier, G., Reneau, J.C., Bazhanov, N., Oh, J.Y., and Prockop,

D.J. (2014). TSG-6 as a biomarker to predict efficacy of human mesenchymal stem/progenitor cells (hMSCs) in modulating sterile inflammation in vivo. Proc Natl Acad Sci U S A *111*, 16766-16771. 10.1073/pnas.1416121111.

105. Lee, S., Choi, E., Cha, M.J., and Hwang, K.C. (2015). Cell adhesion and long-term survival of transplanted mesenchymal stem cells: a prerequisite for cell therapy. Oxid Med Cell Longev *2015*, 632902. 10.1155/2015/632902.

106. Leng, Z., Zhu, R., Hou, W., Feng, Y., Yang, Y., Han, Q., Shan, G., Meng, F., Du, D., Wang, S., et al. (2020). Transplantation of ACE2(-) Mesenchymal Stem Cells Improves the Outcome of Patients with COVID-19 Pneumonia. Aging Dis *11*, 216-228. 10.14336/AD.2020.0228.

107. Leuning, D.G., Beijer, N.R.M., du Fosse, N.A., Vermeulen, S., Lievers, E., van Kooten, C., Rabelink, T.J., and Boer, J. (2018). The cytokine secretion profile of mesenchymal stromal cells is determined by surface structure of the microenvironment. Sci Rep *8*, 7716. 10.1038/s41598-018-25700-5.

108. Li, W., Ren, G., Huang, Y., Su, J., Han, Y., Li, J., Chen, X., Cao, K., Chen, Q., Shou, P., et al. (2012). Mesenchymal stem cells: a double-edged sword in regulating immune responses. Cell Death Differ *19*, 1505-1513. 10.1038/cdd.2012.26.

109. Li, X., Bai, J., Ji, X., Li, R., Xuan, Y., and Wang, Y. (2014). Comprehensive characterization of four different populations of human mesenchymal stem cells as regards their immune properties, proliferation and differentiation. Int J Mol Med *34*, 695-704. 10.3892/ijmm.2014.1821.

110. Li, X., Hong, Y., He, H., Jiang, G., You, W., Liang, X., Fu, Q., Han, S., Lian, Q., and Zhang, Y. (2019). FGF21 Mediates Mesenchymal Stem Cell Senescence via Regulation of Mitochondrial Dynamics. Oxid Med Cell Longev *2019*, 4915149. 10.1155/2019/4915149.

111. Li, X., Yue, S., and Luo, Z. (2017). Mesenchymal stem cells in idiopathic pulmonary fibrosis. Oncotarget *8*, 102600-102616. 10.18632/oncotarget.18126.

112. Lin, S., Lee, W.Y.W., Feng, Q., Xu, L., Wang, B., Man, G.C.W., Chen, Y., Jiang, X., Bian, L., Cui, L., et al. (2017). Synergistic effects on mesenchymal stem cell-based cartilage regeneration by chondrogenic preconditioning and mechanical stimulation. Stem Cell Res Ther *8*, 221. 10.1186/s13287-017-0672-5.

113. Liu, F., Shi, J., Zhang, Y., Lian, A., Han, X., Zuo, K., Liu, M., Zheng, T., Zou, F., Liu, X., et al. (2019). NANOG Attenuates Hair Follicle-Derived Mesenchymal Stem Cell Senescence by Upregulating PBX1 and Activating AKT Signaling. Oxid Med Cell Longev *2019*, 4286213. 10.1155/2019/4286213.

114. Liu, H., Honmou, O., Harada, K., Nakamura, K., Houkin, K., Hamada, H., and Kocsis, J.D. (2006). Neuroprotection by PlGF gene-modified human mesenchymal stem cells after cerebral ischaemia. Brain *129*, 2734-2745. 10.1093/brain/awl207.

115. Liu, M., Lei, H., Dong, P., Fu, X., Yang, Z., Yang, Y., Ma, J., Liu, X., Cao, Y., and Xiao, R. (2017). Adipose-Derived Mesenchymal Stem Cells from the Elderly Exhibit Decreased Migration and Differentiation Abilities with Senescent Properties. Cell Transplant *26*, 1505-1519.

10.1177/0963689717721221.

116. Lo Surdo, J., and Bauer, S.R. (2012). Quantitative approaches to detect donor and passage differences in adipogenic potential and clonogenicity in human bone marrow-derived mesenchymal stem cells. Tissue Eng Part C Methods *18*, 877-889. 10.1089/ten.TEC.2011.0736.

117. Loebel, C., Czekanska, E.M., Bruderer, M., Salzmann, G., Alini, M., and Stoddart, M.J. (2015). In vitro osteogenic potential of human mesenchymal stem cells is predicted by Runx2/Sox9 ratio. Tissue Eng Part A *21*, 115-123. 10.1089/ten.TEA.2014.0096.

118. Lowe, C.E., O'Rahilly, S., and Rochford, J.J. (2011). Adipogenesis at a glance. J Cell Sci *124*, 2681-2686. 10.1242/jcs.079699.

119. Luo, H., Guo, Y., Liu, Y., Wang, Y., Zheng, R., Ban, Y., Peng, L., Yuan, Q., and Liu, W. (2019). Growth differentiation factor 11 inhibits adipogenic differentiation by activating TGF-beta/Smad signalling pathway. Cell Prolif *52*, e12631. 10.1111/cpr.12631.

120. Lv, F.J., Tuan, R.S., Cheung, K.M., and Leung, V.Y. (2014). Concise review: the surface markers and identity of human mesenchymal stem cells. Stem Cells *32*, 1408-1419. 10.1002/stem.1681.

121. Lv, H., Liu, Q., Sun, Y., Yi, X., Wei, X., Liu, W., Zhang, Q., Yi, H., and Chen, G. (2020). Mesenchymal stromal cells ameliorate acute lung injury induced by LPS mainly through stanniocalcin-2 mediating macrophage polarization. Ann Transl Med *8*, 334. 10.21037/atm.2020.02.105.

122. M, H., J, C., H, G., and KW, L. (2008). In vitro chondrogenesis of mesenchymal stem cells in

recombinant silk-elastinlike hydrogels. Pharmaceutical research *25*. 10.1007/s11095-007-9282-8.

123. Ma, S., Xie, N., Li, W., Yuan, B., Shi, Y., and Wang, Y. (2014). Immunobiology of mesenchymal stem cells. Cell Death Differ *21*, 216-225. 10.1038/cdd.2013.158.

124. Maacha, S., Sidahmed, H., Jacob, S., Gentilcore, G., Calzone, R., Grivel, J.C., and Cugno, C. (2020). Paracrine Mechanisms of Mesenchymal Stromal Cells in Angiogenesis. Stem Cells Int *2020*, 4356359. 10.1155/2020/4356359.

125. Madrigal, M., Rao, K.S., and Riordan, N.H. (2014). A review of therapeutic effects of mesenchymal stem cell secretions and induction of secretory modification by different culture methods. J Transl Med *12*, 260. 10.1186/s12967-014-0260-8.

126. Mao, G., Zhang, Z., Hu, S., Zhang, Z., Chang, Z., Huang, Z., Liao, W., and Kang, Y. (2018). Exosomes derived from miR-92a-3p-overexpressing human mesenchymal stem cells enhance chondrogenesis and suppress cartilage degradation via targeting WNT5A. Stem Cell Res Ther *9*, 247. 10.1186/s13287-018-1004-0.

127. Marin-Acevedo, J.A., Dholaria, B., Soyano, A.E., Knutson, K.L., Chumsri, S., and Lou, Y. (2018). Next generation of immune checkpoint therapy in cancer: new developments and challenges. J Hematol Oncol *11*, 39. 10.1186/s13045-018-0582-8.

128. Maroni, G., Tkachuk, V.A., Egorov, A., Morelli, M.J., Luongo, R., Levantini, E., Blasi, F., Magli, M.C., and Penkov, D. (2017). Prep1 prevents premature adipogenesis of mesenchymal progenitors.

Sci Rep *7*, 15573. 10.1038/s41598-017-15828-1.

129. Marquez-Curtis, L.A., and Janowska-Wieczorek, A. (2013). Enhancing the migration ability of mesenchymal stromal cells by targeting the SDF-1/CXCR4 axis. Biomed Res Int *2013*, 561098. 10.1155/2013/561098.

130. McCully, M., Conde, J., P, V.B., Mullin, M., Dalby, M.J., and Berry, C.C. (2018). Nanoparticle-antagomiR based targeting of miR-31 to induce osterix and osteocalcin expression in mesenchymal stem cells. PLoS One *13*, e0192562. 10.1371/journal.pone.0192562.

131. McHugh, D., and Gil, J. (2018). Senescence and aging: Causes, consequences, and therapeutic avenues. J Cell Biol *217*, 65-77. 10.1083/jcb.201708092.

132. Menssen, A., Haupl, T., Sittinger, M., Delorme, B., Charbord, P., and Ringe, J. (2011). Differential gene expression profiling of human bone marrow-derived mesenchymal stem cells during adipogenic development. BMC Genomics *12*, 461. 10.1186/1471-2164-12-461.

133. Mias, C., Lairez, O., Trouche, E., Roncalli, J., Calise, D., Seguelas, M.H., Ordener, C., Piercecchi-Marti, M.D., Auge, N., Salvayre, A.N., et al. (2009). Mesenchymal stem cells promote matrix metalloproteinase secretion by cardiac fibroblasts and reduce cardiac ventricular fibrosis after myocardial infarction. Stem Cells *27*, 2734-2743. 10.1002/stem.169.

134. Miceli, V., Pampalone, M., Vella, S., Carreca, A.P., Amico, G., and Conaldi, P.G. (2019). Comparison of Immunosuppressive and Angiogenic Properties of Human Amnion-Derived Mesenchymal Stem

Cells between 2D and 3D Culture Systems. Stem Cells Int *2019*, 7486279. 10.1155/2019/7486279.

135. Mizukami, A., Thome, C.H., Ferreira, G.A., Lanfredi, G.P., Covas, D.T., Pitteri, S.J., Swiech, K., and Faca, V.M. (2019). Proteomic Identification and Time-Course Monitoring of Secreted Proteins During Expansion of Human Mesenchymal Stem/Stromal in Stirred-Tank Bioreactor. Front Bioeng Biotechnol *7*, 154. 10.3389/fbioe.2019.00154.

136. Mizuno, M., Katano, H., Mabuchi, Y., Ogata, Y., Ichinose, S., Fujii, S., Otabe, K., Komori, K., Ozeki, N., Koga, H., et al. (2018). Specific markers and properties of synovial mesenchymal stem cells in the surface, stromal, and perivascular regions. Stem Cell Res Ther *9*, 123. 10.1186/s13287-018-0870-9.

137. Mochizuki, T., Muneta, T., Sakaguchi, Y., Nimura, A., Yokoyama, A., Koga, H., and Sekiya, I. (2006). Higher chondrogenic potential of fibrous synovium- and adipose synovium-derived cells compared with subcutaneous fat-derived cells: distinguishing properties of mesenchymal stem cells in humans. Arthritis Rheum *54*, 843-853. 10.1002/art.21651.

138. Mueller, M.B., and Tuan, R.S. (2008). Functional characterization of hypertrophy in chondrogenesis of human mesenchymal stem cells. Arthritis Rheum *58*, 1377-1388. 10.1002/art.23370.

139. Mussano, F., Genova, T., Corsalini, M., Schierano, G., Pettini, F., Di Venere, D., and Carossa, S. (2017). Cytokine, Chemokine, and Growth Factor Profile Characterization of Undifferentiated and Osteoinduced Human Adipose-Derived Stem Cells. Stem Cells Int *2017*, 6202783.

10.1155/2017/6202783.

140. Mwale, F., Stachura, D., Roughley, P., and Antoniou, J. (2006). Limitations of using aggrecan and type X collagen as markers of chondrogenesis in mesenchymal stem cell differentiation. J Orthop Res *24*, 1791-1798. 10.1002/jor.20200.

141. Nakagawa, Y., Muneta, T., Otabe, K., Ozeki, N., Mizuno, M., Udo, M., Saito, R., Yanagisawa, K., Ichinose, S., Koga, H., et al. (2016). Cartilage Derived from Bone Marrow Mesenchymal Stem Cells Expresses Lubricin In Vitro and In Vivo. PLoS One *11*, e0148777. 10.1371/journal.pone.0148777.

142. Nakanishi, C., Nagaya, N., Ohnishi, S., Yamahara, K., Takabatake, S., Konno, T., Hayashi, K., Kawashiri, M.A., Tsubokawa, T., and Yamagishi, M. (2011). Gene and protein expression analysis of mesenchymal stem cells derived from rat adipose tissue and bone marrow. Circ J *75*, 2260-2268. 10.1253/circj.cj-11-0246.

143. Nemeth, K., Leelahavanichkul, A., Yuen, P.S., Mayer, B., Parmelee, A., Doi, K., Robey, P.G., Leelahavanichkul, K., Koller, B.H., Brown, J.M., et al. (2009). Bone marrow stromal cells attenuate sepsis via prostaglandin E(2)-dependent reprogramming of host macrophages to increase their interleukin-10 production. Nat Med *15*, 42-49. 10.1038/nm.1905.

144. Neri, S., and Borzi, R.M. (2020). Molecular Mechanisms Contributing to Mesenchymal Stromal Cell Aging. Biomolecules *10*. 10.3390/biom10020340.

145. Newman, R.E., Yoo, D., LeRoux, M.A., and Danilkovitch-Miagkova, A. (2009). Treatment of

inflammatory diseases with mesenchymal stem cells. Inflamm Allergy Drug Targets *8*, 110-123. 10.2174/187152809788462635.

146. Nishizawa, K., and Seki, R. (2016). Mechanisms of immunosuppression by mesenchymal stromal cells: a review with a focus on molecules. Biomedical Research and Clinical Practice *1*. 10.15761/brcp.1000116.

147. Nitzsche, F., Muller, C., Lukomska, B., Jolkkonen, J., Deten, A., and Boltze, J. (2017). Concise Review: MSC Adhesion Cascade-Insights into Homing and Transendothelial Migration. Stem Cells *35*, 1446-1460. 10.1002/stem.2614.

148. Noiseux, N., Borie, M., Desnoyers, A., Menaouar, A., Stevens, L.M., Mansour, S., Danalache, B.A., Roy, D.C., Jankowski, M., and Gutkowska, J. (2012). Preconditioning of stem cells by oxytocin to improve their therapeutic potential. Endocrinology *153*, 5361-5372. 10.1210/en.2012-1402.

149. Oberbauer, E., Steffenhagen, C., Feichtinger, G., Hildner, F., Hacobian, A., Danzer, M., Gabriel, C., Redl, H., and Wolbank, S. (2016). A Luciferase-Based Quick Potency Assay to Predict Chondrogenic Differentiation. Tissue Eng Part C Methods *22*, 487-495. 10.1089/ten.TEC.2015.0435.

150. Ogata, Y., Mabuchi, Y., Yoshida, M., Suto, E.G., Suzuki, N., Muneta, T., Sekiya, I., and Akazawa, C. (2015). Purified Human Synovium Mesenchymal Stem Cells as a Good Resource for Cartilage Regeneration. PLoS One *10*, e0129096. 10.1371/journal.pone.0129096.

151. Oh, J.Y., Lee, R.H., Yu, J.M., Ko, J.H., Lee, H.J., Ko, A.Y., Roddy, G.W., and Prockop, D.J. (2012).

Intravenous mesenchymal stem cells prevented rejection of allogeneic corneal transplants by aborting the early inflammatory response. Mol Ther *20*, 2143-2152. 10.1038/mt.2012.165.

152. Oskowitz, A., McFerrin, H., Gutschow, M., Carter, M.L., and Pochampally, R. (2011). Serum-deprived human multipotent mesenchymal stromal cells (MSCs) are highly angiogenic. Stem Cell Res *6*, 215-225. 10.1016/j.scr.2011.01.004.

153. Ozeki, N., Muneta, T., Koga, H., Nakagawa, Y., Mizuno, M., Tsuji, K., Mabuchi, Y., Akazawa, C., Kobayashi, E., Matsumoto, K., et al. (2016). Not single but periodic injections of synovial mesenchymal stem cells maintain viable cells in knees and inhibit osteoarthritis progression in rats. Osteoarthritis Cartilage *24*, 1061-1070. 10.1016/j.joca.2015.12.018.

154. P, D.G., C, L., M, B., A, T., E, F., R, V., C, P., M, P., and A, F. (2006). A real-time PCR approach to evaluate adipogenic potential of amniotic fluid-derived human mesenchymal stem cells. Stem cells and development *15*. 10.1089/scd.2006.15.719.

155. P, M., S, H., R, M., M, G., and W, S.K. (2011). Adult mesenchymal stem cells and cell surface characterization - a systematic review of the literature. Open Orthop J *5*, 253-260. 10.2174/1874325001105010253.

156. Pardoll, D.M. (2012). The blockade of immune checkpoints in cancer immunotherapy. Nat Rev Cancer *12*, 252-264. 10.1038/nrc3239.

157. Park, C.W., Kim, K.S., Bae, S., Son, H.K., Myung, P.K., Hong, H.J., and Kim, H. (2009). Cytokine

secretion profiling of human mesenchymal stem cells by antibody array. Int J Stem Cells *2*, 59-68. 10.15283/ijsc.2009.2.1.59.

158. Park, H.S., Ashour, D., Elsharoud, A., Chugh, R.M., Ismail, N., El Andaloussi, A., and Al-Hendy, A. (2019). Towards Cell free Therapy of Premature Ovarian Insufficiency: Human Bone Marrow Mesenchymal Stem Cells Secretome Enhances Angiogenesis in Human Ovarian Microvascular Endothelial Cells. HSOA J Stem Cells Res Dev Ther *5*. 10.24966/srdt-2060/100019.

159. Pittenger, M.F., Discher, D.E., Peault, B.M., Phinney, D.G., Hare, J.M., and Caplan, A.I. (2019). Mesenchymal stem cell perspective: cell biology to clinical progress. NPJ Regen Med *4*, 22. 10.1038/s41536-019-0083-6.

160. Pleumeekers, M.M., Nimeskern, L., Koevoet, J.L.M., Karperien, M., Stok, K.S., and van Osch, G. (2018). Trophic effects of adipose-tissue-derived and bone-marrow-derived mesenchymal stem cells enhance cartilage generation by chondrocytes in co-culture. PLoS One *13*, e0190744. 10.1371/journal.pone.0190744.

161. Poggi, A., and Giuliani, M. (2016). Mesenchymal Stromal Cells Can Regulate the Immune Response in the Tumor Microenvironment. Vaccines (Basel) *4*. 10.3390/vaccines4040041.

162. Pokrovskaya, L.A., Zubareva, E.V., Nadezhdin, S.V., Lysenko, A.S., and Litovkina, T.L. (2020). Biological activity of mesenchymal stem cells secretome as a basis for cell-free therapeutic approach. Research Results in Pharmacology *6*, 57-68. 10.3897/rrpharmacology.6.49413.

163. Pokrywczynska, M., Maj, M., Kloskowski, T., Buhl, M., Balcerczyk, D., Jundzill, A., Szeliski, K., Rasmus, M., and Drewa, T. (2020). Molecular Aspects of Adipose-Derived Stromal Cell Senescence in a Long-Term Culture: A Potential Role of Inflammatory Pathways. Cell Transplant *29*, 963689720917341. 10.1177/0963689720917341.

164. Ponte, A.L., Marais, E., Gallay, N., Langonne, A., Delorme, B., Herault, O., Charbord, P., and Domenech, J. (2007). The in vitro migration capacity of human bone marrow mesenchymal stem cells: comparison of chemokine and growth factor chemotactic activities. Stem Cells *25*, 1737-1745. 10.1634/stemcells.2007-0054.

165. Popov, C., Radic, T., Haasters, F., Prall, W.C., Aszodi, A., Gullberg, D., Schieker, M., and Docheva, D. (2011). Integrins alpha2beta1 and alpha11beta1 regulate the survival of mesenchymal stem cells on collagen I. Cell Death Dis *2*, e186. 10.1038/cddis.2011.71.

166. Prockop, D.J., and Oh, J.Y. (2012). Mesenchymal stem/stromal cells (MSCs): role as guardians of inflammation. Mol Ther *20*, 14-20. 10.1038/mt.2011.211.

167. Qi, X., Zhang, J., Yuan, H., Xu, Z., Li, Q., Niu, X., Hu, B., Wang, Y., and Li, X. (2016). Exosomes Secreted by Human-Induced Pluripotent Stem Cell-Derived Mesenchymal Stem Cells Repair Critical-Sized Bone Defects through Enhanced Angiogenesis and Osteogenesis in Osteoporotic Rats. Int J Biol Sci *12*, 836-849. 10.7150/ijbs.14809.

168. Qiong, J., Xia, Z., Jing, L., and Haibin, W. (2020). Synovial mesenchymal stem cells effectively

alleviate osteoarthritis through promoting the proliferation and differentiation of meniscus chondrocytes. Eur Rev Med Pharmacol Sci *24*, 1645-1655. 10.26355/eurrev_202002_20338.

169. Rahbarghazi, J.R.M.H.M.H.H.A.E.S.M.A.R.R.J.R.M.H.M.H.H.A.E.S.M.A.R. (2019). The Angiogenic Paracrine Potential of Mesenchymal Stem Cells 10.5772/intechopen.84433.

170. Rakic, R., Bourdon, B., Demoor, M., Maddens, S., Saulnier, N., and Galera, P. (2018). Differences in the intrinsic chondrogenic potential of equine umbilical cord matrix and cord blood mesenchymal stromal/stem cells for cartilage regeneration. Sci Rep *8*, 13799. 10.1038/s41598-018-28164-9.

171. Ratushnyy, A., Ezdakova, M., and Buravkova, L. (2020). Secretome of Senescent Adipose-Derived Mesenchymal Stem Cells Negatively Regulates Angiogenesis. Int J Mol Sci *21*. 10.3390/ijms21051802.

172. Reinders, M.E., and Hoogduijn, M.J. (2014). NK Cells and MSCs: Possible Implications for MSC Therapy in Renal Transplantation. J Stem Cell Res Ther *4*, 1000166. 10.4172/2157-7633.1000166.

173. Ren, G., Zhang, L., Zhao, X., Xu, G., Zhang, Y., Roberts, A.I., Zhao, R.C., and Shi, Y. (2008). Mesenchymal stem cell-mediated immunosuppression occurs via concerted action of chemokines and nitric oxide. Cell Stem Cell *2*, 141-150. 10.1016/j.stem.2007.11.014.

174. Ren, G., Zhao, X., Zhang, L., Zhang, J., L'Huillier, A., Ling, W., Roberts, A.I., Le, A.D., Shi, S., Shao, C., and Shi, Y. (2010). Inflammatory cytokine-induced intercellular adhesion molecule-1 and vascular cell adhesion molecule-1 in mesenchymal stem cells are critical for immunosuppression. J Immunol

184, 2321-2328. 10.4049/jimmunol.0902023.

175. Rengasamy, M., Singh, G., Fakharuzi, N.A., Siddikuzzaman, Balasubramanian, S., Swamynathan, P., Thej, C., Sasidharan, G., Gupta, P.K., Das, A.K., et al. (2017). Transplantation of human bone marrow mesenchymal stromal cells reduces liver fibrosis more effectively than Wharton's jelly mesenchymal stromal cells. Stem Cell Res Ther *8*, 143. 10.1186/s13287-017-0595-1.

176. Ribeiro, T.O., Silveira, B.M., Meira, M.C., Carreira, A.C.O., Sogayar, M.C., Meyer, R., and Fortuna, V. (2019). Investigating the potential of the secretome of mesenchymal stem cells derived from sickle cell disease patients. PLoS One *14*, e0222093. 10.1371/journal.pone.0222093.

177. Riekstina, U., Cakstina, I., Parfejevs, V., Hoogduijn, M., Jankovskis, G., Muiznieks, I., Muceniece, R., and Ancans, J. (2009). Embryonic stem cell marker expression pattern in human mesenchymal stem cells derived from bone marrow, adipose tissue, heart and dermis. Stem Cell Rev Rep *5*, 378-386. 10.1007/s12015-009-9094-9.

178. RK, J., N, J., SP, B., G, M., DR, M., and MF, P. (2000). Adult human mesenchymal stem cell differentiation to the osteogenic or adipogenic lineage is regulated by mitogen-activated protein kinase. The Journal of biological chemistry *275*. 10.1074/jbc.275.13.9645.

179. Roddy, G.W., Oh, J.Y., Lee, R.H., Bartosh, T.J., Ylostalo, J., Coble, K., Rosa, R.H., Jr., and Prockop, D.J. (2011). Action at a distance: systemically administered adult stem/progenitor cells (MSCs) reduce inflammatory damage to the cornea without engraftment and primarily by secretion of TNF-alpha

stimulated gene/protein 6. Stem Cells *29*, 1572-1579. 10.1002/stem.708.

180. Rogers, C.J., Harman, R.J., Bunnell, B.A., Schreiber, M.A., Xiang, C., Wang, F.S., Santidrian, A.F., and Minev, B.R. (2020). Rationale for the clinical use of adipose-derived mesenchymal stem cells for COVID-19 patients. J Transl Med *18*, 203. 10.1186/s12967-020-02380-2.

181. Romieu-Mourez, R., Francois, M., Boivin, M.N., Bouchentouf, M., Spaner, D.E., and Galipeau, J. (2009). Cytokine modulation of TLR expression and activation in mesenchymal stromal cells leads to a proinflammatory phenotype. J Immunol *182*, 7963-7973. 10.4049/jimmunol.0803864.

182. Rossi, J.F., Ceballos, P., and Lu, Z.Y. (2019). Immune precision medicine for cancer: a novel insight based on the efficiency of immune effector cells. Cancer Commun (Lond) *39*, 34. 10.1186/s40880-019-0379-3.

183. Sakaguchi, Y., Sekiya, I., Yagishita, K., and Muneta, T. (2005). Comparison of human stem cells derived from various mesenchymal tissues: superiority of synovium as a cell source. Arthritis Rheum *52*, 2521-2529. 10.1002/art.21212.

184. Salama, R., Sadaie, M., Hoare, M., and Narita, M. (2014). Cellular senescence and its effector programs. Genes Dev *28*, 99-114. 10.1101/gad.235184.113.

185. Sanchez, D.N.R., Bertanha, M., Fernandes, T.D., Resende, L.A.L., Deffune, E., and Amorim, R.M. (2017). Effects of Canine and Murine Mesenchymal Stromal Cell Transplantation on Peripheral Nerve Regeneration. Int J Stem Cells *10*, 83-92. 10.15283/ijsc16037.

186. Sane, M.S., Misra, N., Mousa, O.M., Czop, S., Tang, H., Khoo, L.T., Jones, C.D., and Mustafi, S.B. (2018). Cytokines in umbilical cord blood-derived cellular product: a mechanistic insight into bone repair. Regen Med *13*, 881-898. 10.2217/rme-2018-0102.

187. Sasaki, M., Radtke, C., Tan, A.M., Zhao, P., Hamada, H., Houkin, K., Honmou, O., and Kocsis, J.D. (2009). BDNF-hypersecreting human mesenchymal stem cells promote functional recovery, axonal sprouting, and protection of corticospinal neurons after spinal cord injury. J Neurosci *29*, 14932-14941. 10.1523/JNEUROSCI.2769-09.2009.

188. Schallmoser, K., Bartmann, C., Rohde, E., Bork, S., Guelly, C., Obenauf, A.C., Reinisch, A., Horn, P., Ho, A.D., Strunk, D., and Wagner, W. (2010). Replicative senescence-associated gene expression changes in mesenchymal stromal cells are similar under different culture conditions. Haematologica *95*, 867-874. 10.3324/haematol.2009.011692.

189. Schutze, N., Noth, U., Schneidereit, J., Hendrich, C., and Jakob, F. (2005). Differential expression of CCN-family members in primary human bone marrow-derived mesenchymal stem cells during osteogenic, chondrogenic and adipogenic differentiation. Cell Commun Signal *3*, 5. 10.1186/1478-811X-3-5.

190. Segers, V.F., Van Riet, I., Andries, L.J., Lemmens, K., Demolder, M.J., De Becker, A.J., Kockx, M.M., and De Keulenaer, G.W. (2006). Mesenchymal stem cell adhesion to cardiac microvascular endothelium: activators and mechanisms. Am J Physiol Heart Circ Physiol *290*, H1370-1377.

10.1152/ajpheart.00523.2005.

191. Sekiya, I., Ojima, M., Suzuki, S., Yamaga, M., Horie, M., Koga, H., Tsuji, K., Miyaguchi, K., Ogishima, S., Tanaka, H., and Muneta, T. (2012). Human mesenchymal stem cells in synovial fluid increase in the knee with degenerated cartilage and osteoarthritis. J Orthop Res *30*, 943-949. 10.1002/jor.22029.

192. Shibata, K.R., Aoyama, T., Shima, Y., Fukiage, K., Otsuka, S., Furu, M., Kohno, Y., Ito, K., Fujibayashi, S., Neo, M., et al. (2007). Expression of the p16INK4A gene is associated closely with senescence of human mesenchymal stem cells and is potentially silenced by DNA methylation during in vitro expansion. Stem Cells *25*, 2371-2382. 10.1634/stemcells.2007-0225.

193. Sofia, V., Bachri, M.S., and Endrinaldi, E. (2019). The Influence of Mesenchymal Stem Cell Wharton Jelly toward Prostaglandin E2 Gene Expression on Synoviocyte Cell Osteoarthritis. Open Access Maced J Med Sci *7*, 1252-1258. 10.3889/oamjms.2019.082.

194. Sohni, A., and Verfaillie, C.M. (2013). Mesenchymal stem cells migration homing and tracking. Stem Cells Int *2013*, 130763. 10.1155/2013/130763.

195. Somaiah, C., Kumar, A., Mawrie, D., Sharma, A., Patil, S.D., Bhattacharyya, J., Swaminathan, R., and Jaganathan, B.G. (2015). Collagen Promotes Higher Adhesion, Survival and Proliferation of Mesenchymal Stem Cells. PLoS One *10*, e0145068. 10.1371/journal.pone.0145068.

196. Song, P., Han, T., Xiang, X., Wang, Y., Fang, H., Niu, Y., and Shen, C. (2020). The role of hepatocyte

growth factor in mesenchymal stem cell-induced recovery in spinal cord injured rats. Stem Cell Res Ther *11*, 178. 10.1186/s13287-020-01691-x.

197. Sordi, V., Malosio, M.L., Marchesi, F., Mercalli, A., Melzi, R., Giordano, T., Belmonte, N., Ferrari, G., Leone, B.E., Bertuzzi, F., et al. (2005). Bone marrow mesenchymal stem cells express a restricted set of functionally active chemokine receptors capable of promoting migration to pancreatic islets. Blood *106*, 419-427. 10.1182/blood-2004-09-3507.

198. Spees, J.L., Lee, R.H., and Gregory, C.A. (2016). Mechanisms of mesenchymal stem/stromal cell function. Stem Cell Res Ther *7*, 125. 10.1186/s13287-016-0363-7.

199. Studle, C., Occhetta, P., Geier, F., Mehrkens, A., Barbero, A., and Martin, I. (2019). Challenges Toward the Identification of Predictive Markers for Human Mesenchymal Stromal Cells Chondrogenic Potential. Stem Cells Transl Med *8*, 194-204. 10.1002/sctm.18-0147.

200. Sun, C., Wang, L., Wang, H., Huang, T., Yao, W., Li, J., and Zhang, X. (2020). Single-cell RNA-seq highlights heterogeneity in human primary Wharton's jelly mesenchymal stem/stromal cells cultured in vitro. Stem Cell Res Ther *11*, 149. 10.1186/s13287-020-01660-4.

201. Sung, D.K., Chang, Y.S., Ahn, S.Y., Sung, S.I., Yoo, H.S., Choi, S.J., Kim, S.Y., and Park, W.S. (2015). Optimal Route for Human Umbilical Cord Blood-Derived Mesenchymal Stem Cell Transplantation to Protect Against Neonatal Hyperoxic Lung Injury: Gene Expression Profiles and Histopathology. PLoS One *10*, e0135574. 10.1371/journal.pone.0135574.

202. Tao, H., Han, Z., Han, Z.C., and Li, Z. (2016). Proangiogenic Features of Mesenchymal Stem Cells and Their Therapeutic Applications. Stem Cells Int *2016*, 1314709. 10.1155/2016/1314709.

203. Tebebi, P.A., Kim, S.J., Williams, R.A., Milo, B., Frenkel, V., Burks, S.R., and Frank, J.A. (2017). Improving the therapeutic efficacy of mesenchymal stromal cells to restore perfusion in critical limb ischemia through pulsed focused ultrasound. Sci Rep *7*, 41550. 10.1038/srep41550.

204. Teixeira, F.G., Carvalho, M.M., Panchalingam, K.M., Rodrigues, A.J., Mendes-Pinheiro, B., Anjo, S., Manadas, B., Behie, L.A., Sousa, N., and Salgado, A.J. (2017). Impact of the Secretome of Human Mesenchymal Stem Cells on Brain Structure and Animal Behavior in a Rat Model of Parkinson's Disease. Stem Cells Transl Med *6*, 634-646. 10.5966/sctm.2016-0071.

205. Terunuma, A., Ashiba, K., Takane, T., Sakaguchi, Y., and Terunuma, H. (2019). Comparative transcriptomic analysis of human mesenchymal stem cells derived from dental pulp and adipose tissues. J Stem Cells Regen Med *15*, 8-11. 10.46582/jsrm.1501003.

206. Tofino-Vian, M., Guillen, M.I., Perez Del Caz, M.D., Silvestre, A., and Alcaraz, M.J. (2018). Microvesicles from Human Adipose Tissue-Derived Mesenchymal Stem Cells as a New Protective Strategy in Osteoarthritic Chondrocytes. Cell Physiol Biochem *47*, 11-25. 10.1159/000489739.

207. Tsai, M.T., Li, W.J., Tuan, R.S., and Chang, W.H. (2009). Modulation of osteogenesis in human mesenchymal stem cells by specific pulsed electromagnetic field stimulation. J Orthop Res *27*, 1169-1174. 10.1002/jor.20862.

208. Tucker, D., Still, K., Blom, A., Hollander, A.P., and Kafienah, W. (2020). Over-Confluence of expanded bone marrow mesenchymal stem cells ameliorates their chondrogenic capacity in 3D cartilage tissue engineering. bioRxiv. 10.1101/2020.01.08.897645.

209. Turinetto, V., Vitale, E., and Giachino, C. (2016). Senescence in Human Mesenchymal Stem Cells: Functional Changes and Implications in Stem Cell-Based Therapy. Int J Mol Sci *17*. 10.3390/ijms17071164.

210. Ullah, M., Liu, D.D., and Thakor, A.S. (2019). Mesenchymal Stromal Cell Homing: Mechanisms and Strategies for Improvement. iScience *15*, 421-438. 10.1016/j.isci.2019.05.004.

211. Usunier, B., Benderitter, M., Tamarat, R., and Chapel, A. (2014). Management of fibrosis: the mesenchymal stromal cells breakthrough. Stem Cells Int *2014*, 340257. 10.1155/2014/340257.

212. van Buul, G.M., Villafuertes, E., Bos, P.K., Waarsing, J.H., Kops, N., Narcisi, R., Weinans, H., Verhaar, J.A., Bernsen, M.R., and van Osch, G.J. (2012). Mesenchymal stem cells secrete factors that inhibit inflammatory processes in short-term osteoarthritic synovium and cartilage explant culture. Osteoarthritis Cartilage *20*, 1186-1196. 10.1016/j.joca.2012.06.003.

213. Vanden Berg-Foels, W.S. (2014). In situ tissue regeneration: chemoattractants for endogenous stem cell recruitment. Tissue Eng Part B Rev *20*, 28-39. 10.1089/ten.TEB.2013.0100.

214. W, W., S, B., G, L., S, J., N, M., D, S., and C, K. (2010). How to track cellular aging of mesenchymal stromal cells? Aging *2*. 10.18632/aging.100136.

215. Wagner, W., Horn, P., Castoldi, M., Diehlmann, A., Bork, S., Saffrich, R., Benes, V., Blake, J., Pfister, S., Eckstein, V., and Ho, A.D. (2008). Replicative senescence of mesenchymal stem cells: a continuous and organized process. PLoS One *3*, e2213. 10.1371/journal.pone.0002213.

216. Wang, A.Y.L., Loh, C.Y.Y., Shen, H.H., Hsieh, S.Y., Wang, I.K., Chuang, S.H., and Wei, F.C. (2019). Topical Application of Human Wharton's Jelly Mesenchymal Stem Cells Accelerates Mouse Sciatic Nerve Recovery and is Associated with Upregulated Neurotrophic Factor Expression. Cell Transplant *28*, 1560-1572. 10.1177/0963689719880543.

217. Wang, M., Yuan, Q., and Xie, L. (2018). Mesenchymal Stem Cell-Based Immunomodulation: Properties and Clinical Application. Stem Cells Int *2018*, 3057624. 10.1155/2018/3057624.

218. Watt, S.M., Gullo, F., van der Garde, M., Markeson, D., Camicia, R., Khoo, C.P., and Zwaginga, J.J. (2013). The angiogenic properties of mesenchymal stem/stromal cells and their therapeutic potential. Br Med Bull *108*, 25-53. 10.1093/bmb/ldt031.

219. Weiss, A.R.R., and Dahlke, M.H. (2019). Immunomodulation by Mesenchymal Stem Cells (MSCs): Mechanisms of Action of Living, Apoptotic, and Dead MSCs. Front Immunol *10*, 1191. 10.3389/fimmu.2019.01191.

220. Weiss, M.L., Medicetty, S., Bledsoe, A.R., Rachakatla, R.S., Choi, M., Merchav, S., Luo, Y., Rao, M.S., Velagaleti, G., and Troyer, D. (2006). Human umbilical cord matrix stem cells: preliminary characterization and effect of transplantation in a rodent model of Parkinson's disease. Stem Cells *24*,

781-792. 10.1634/stemcells.2005-0330.

221. Wiese, D.M., Ruttan, C.C., Wood, C.A., Ford, B.N., and Braid, L.R. (2019). Accumulating Transcriptome Drift Precedes Cell Aging in Human Umbilical Cord-Derived Mesenchymal Stromal Cells Serially Cultured to Replicative Senescence. Stem Cells Transl Med *8*, 945-958. 10.1002/sctm.18-0246.

222. Wu, G.H., Shi, H.J., Che, M.T., Huang, M.Y., Wei, Q.S., Feng, B., Ma, Y.H., Wang, L.J., Jiang, B., Wang, Y.Q., et al. (2018). Recovery of paralyzed limb motor function in canine with complete spinal cord injury following implantation of MSC-derived neural network tissue. Biomaterials *181*, 15-34. 10.1016/j.biomaterials.2018.07.010.

223. Wu, K.C., Chang, Y.H., Liu, H.W., and Ding, D.C. (2019). Transplanting human umbilical cord mesenchymal stem cells and hyaluronate hydrogel repairs cartilage of osteoarthritis in the minipig model. Ci Ji Yi Xue Za Zhi *31*, 11-19. 10.4103/tcmj.tcmj_87_18.

224. Wykes, M.N., and Lewin, S.R. (2018). Immune checkpoint blockade in infectious diseases. Nat Rev Immunol *18*, 91-104. 10.1038/nri.2017.112.

225. Xu, F., Jin, T., Zhu, Y., and Dai, C. (2018). Immune checkpoint therapy in liver cancer. J Exp Clin Cancer Res *37*, 110. 10.1186/s13046-018-0777-4.

226. Xu, J., Wang, W., Ludeman, M., Cheng, K., Hayami, T., Lotz, J.C., and Kapila, S. (2008). Chondrogenic differentiation of human mesenchymal stem cells in three-dimensional alginate gels.

Tissue Eng Part A *14*, 667-680. 10.1089/tea.2007.0272.

227. YA, R., AN, D., NV, M., and LB, B. (2005). Mesenchymal stem cells from human bone marrow and adipose tissue: isolation, characterization, and differentiation potentialities. Bulletin of experimental biology and medicine *140*. 10.1007/s10517-005-0430-z.

228. Yagi, H., Soto-Gutierrez, A., Parekkadan, B., Kitagawa, Y., Tompkins, R.G., Kobayashi, N., and Yarmush, M.L. (2010). Mesenchymal stem cells: Mechanisms of immunomodulation and homing. Cell Transplant *19*, 667-679. 10.3727/096368910X508762.

229. Yang, D.C., Yang, M.H., Tsai, C.C., Huang, T.F., Chen, Y.H., and Hung, S.C. (2011). Hypoxia inhibits osteogenesis in human mesenchymal stem cells through direct regulation of RUNX2 by TWIST. PLoS One *6*, e23965. 10.1371/journal.pone.0023965.

230. Yang, Y., Lin, H., Shen, H., Wang, B., Lei, G., and Tuan, R.S. (2018). Mesenchymal stem cell-derived extracellular matrix enhances chondrogenic phenotype of and cartilage formation by encapsulated chondrocytes in vitro and in vivo. Acta Biomater *69*, 71-82. 10.1016/j.actbio.2017.12.043.

231. Yao, Y., Deng, Q., Song, W., Zhang, H., Li, Y., Yang, Y., Fan, X., Liu, M., Shang, J., Sun, C., et al. (2016). MIF Plays a Key Role in Regulating Tissue-Specific Chondro-Osteogenic Differentiation Fate of Human Cartilage Endplate Stem Cells under Hypoxia. Stem Cell Reports *7*, 249-262. 10.1016/j.stemcr.2016.07.003.

232. Yoshida, K., Nakashima, A., Doi, S., Ueno, T., Okubo, T., Kawano, K.I., Kanawa, M., Kato, Y.,

Higashi, Y., and Masaki, T. (2018). Serum-Free Medium Enhances the Immunosuppressive and Antifibrotic Abilities of Mesenchymal Stem Cells Utilized in Experimental Renal Fibrosis. Stem Cells Transl Med *7*, 893-905. 10.1002/sctm.17-0284.

233. Yu, K.R., and Kang, K.S. (2013). Aging-related genes in mesenchymal stem cells: a mini-review. Gerontology *59*, 557-563. 10.1159/000353857.

234. Yu, S., Zhao, Y., Ma, Y., and Ge, L. (2016). Profiling the Secretome of Human Stem Cells from Dental Apical Papilla. Stem Cells Dev *25*, 499-508. 10.1089/scd.2015.0298.

235. Z, W., and HW, B. (2019). Cnidium lactone stimulates osteogenic differentiation of bone marrow mesenchymal stem cells via BMP-2/smad-signaling cascades mediated by estrogen receptor. American journal of translational research *11*.

236. Zhang, S., Chuah, S.J., Lai, R.C., Hui, J.H.P., Lim, S.K., and Toh, W.S. (2018). MSC exosomes mediate cartilage repair by enhancing proliferation, attenuating apoptosis and modulating immune reactivity. Biomaterials *156*, 16-27. 10.1016/j.biomaterials.2017.11.028.

237. Zhang, T., Lee, Y.W., Rui, Y.F., Cheng, T.Y., Jiang, X.H., and Li, G. (2013). Bone marrow-derived mesenchymal stem cells promote growth and angiogenesis of breast and prostate tumors. Stem Cell Res Ther *4*, 70. 10.1186/scrt221.

238. Zhou, S., Greenberger, J.S., Epperly, M.W., Goff, J.P., Adler, C., Leboff, M.S., and Glowacki, J. (2008). Age-related intrinsic changes in human bone-marrow-derived mesenchymal stem cells and their

differentiation to osteoblasts. Aging Cell *7*, 335-343. 10.1111/j.1474-9726.2008.00377.x.

239. Zhu, Y., Zhang, X., Gu, R., Liu, X., Wang, S., Xia, D., Li, Z., Lian, X., Zhang, P., Liu, Y., and Zhou, Y. (2020). LAMA2 regulates the fate commitment of mesenchymal stem cells via hedgehog signaling. Stem Cell Res Ther *11*, 135. 10.1186/s13287-020-01631-9.

240. Zwolanek, D., Flicker, M., Kirstatter, E., Zaucke, F., van Osch, G.J., and Erben, R.G. (2015). beta1 Integrins Mediate Attachment of Mesenchymal Stem Cells to Cartilage Lesions. Biores Open Access *4*, 39-53. 10.1089/biores.2014.0055.
